# A Volatile Cue From a Specialist Herbivore Primes Gene Expression Against Biotic Stress in Tall Goldenrod (*Solidago altissima* L.)

**DOI:** 10.1111/pce.70279

**Published:** 2025-11-30

**Authors:** Robert J. Witkowski, Lily A. Sudol, Eric C. Yip, John F. Tooker, Tanya Renner

**Affiliations:** ^1^ The Intercollege Graduate Degree Program in Plant Biology, Huck Institutes of Life Science The Pennsylvania State University University Park Pennsylvania USA; ^2^ Department of Entomology The Pennsylvania State University University Park Pennsylvania USA; ^3^ Division of Biological Sciences University of Georgia Athens Georgia USA

**Keywords:** chemical ecology, *Eurosta solidaginis* (goldenrod gall fly), plant defence, priming, *Solidago altissima* (tall goldenrod), transcriptomics

## Abstract

Insect‐derived molecular cues can prime plant defences against herbivore attack. The genes that are sensitive to priming, and how their expression changes on the scale of days, have not been fully resolved. Moreover, priming may affect interactions with insects that are not the source of the priming cue. We primed tall goldenrod (*Solidago altissima*) plants by exposure to the volatile emission of a specialist herbivore, the goldenrod gall fly (*Eurosta solidaginis*) then subjected the plants to 48 h of herbivory from an unrelated generalist, corn earworm (*Helicoverpa zea*). Using RNA sequencing, we identified transcriptome‐wide gene expression patterns between exposed and unexposed plants. We identified biotic stress‐associated genes that were more abundant during herbivory in primed plants, including defence‐related transcription factors, thaumatin‐like receptors and chitinases. We observed a surprising rise and fall in expression of hundreds of defence‐related genes in a 48‐h phase in primed damaged plants only. Our results support the hypothesis that primed defences are stronger than typical induced defences and suggest that primed defences target herbivores in the short term. We show that the threat cue from a specialist can affect plant defences against an unrelated herbivore.

## Summary statement

Plants can detect airborne compounds emitted by insects, which signals that a threat is nearby—these cues ‘prime’ the plants’ defence. We found that if plants detect a threat cue from one insect, they show heightened defences even against an unrelated generalist insect.

## 1 Introduction

Plants can adjust their growth and defence in response to chemical stimuli from their environment. Certain cues, like insect‐ or pathogen‐associated volatile organic compounds (VOCs), can indicate likely sources of future stress and ‘prime’ plant defences in preparation for future challenges, such as herbivory (Frost, Mescher, Carlson, et al. 2008). Primed defences deploy faster or stronger than responses from plants that were not exposed to the priming stimulus (i.e., ‘naïve’ plants; Hilker et al. 2016). Priming theoretically requires anticipatory investment in molecular resources, but evidence of this is scant (Hilker and Schmülling 2019). Such preparatory molecular investments for increased defence should offset greater costs of defence when plants are unprepared for herbivore attacks (Martinez‐Medina et al. 2016). However, the temporal extent of this advantage, that is, if primed defences persist days after triggering, has not been well resolved (Karban 2011). Moreover, VOC‐mediated primed defences likely involve complex regulatory networks of signalling, transcription factors (TFs) and metabolites (Erb 2018), and vary depending on sources of priming cues and subsequent challenges (Kessler and Mueller 2024).

In previous studies, primed responses to herbivory have typically been characterised via increases in, or interplays of, defence‐related hormones (e.g., jasmonic acid [JA], salicylic acid [SA], ethylene [ET] and abscisic acid [ABA], among others; Hilker and Schmülling 2019). For example, green leaf volatiles (GLVs) released from insect‐damaged plants can prime a more intense JA induction upon subsequent feeding (Engelberth et al. 2004; Frost, Mescher, Dervinis, et al. 2008). Moreover, rapid induction of these defence‐related hormones may be caused by accumulation of JA‐related TFs after plants perceive priming cues (Hilker and Schmülling 2019); however, such responses have most frequently been studied via expression of a few (but important) marker genes on the scale of one to 6 h following single bouts of herbivory (Engelberth et al. 2007; Machado et al. 2016; Ye et al. 2019). Therefore, to better understand the underlying genetics and temporal gene expression patterns associated with primed defences, studies should consider a broader array of defence‐related genes over longer timeframes.

Besides herbivory itself, insect‐derived volatile cues can play important but under‐explored roles in mediating plant defences. Cotton plants (*Gossypium hirsutum*) can sense the aggregation pheromone of boll weevil and respond by releasing VOCs to attract their natural enemies (Magalhães et al. 2019), and exposure to the sex pheromone of pine sawfly can prime defence gene expression in Scotch pine (*Pinus sylvestris* L.) upon subsequent egg deposition (Bittner et al. 2019). In addition, recent studies have identified defence priming in tall goldenrod (*Solidago altissima* L., Asteraceae: Astereae) after exposure to the putative sex pheromone emitted by male goldenrod gall fly (*Eurosta solidaginis* Fitch, Diptera: Tephreitidae) (Helms et al. 2013, 2014, 2017; Yip et al. 2017, 2021, 2025). *S*. *altissima* is sensitive to the full blend of the putative pheromone, as well as the most abundant compound in the emission, *E*,*S*‐conophthorin (Helms et al. 2017). While perched on *S. altissima*, *E*. *solidaginis* males call for females by releasing their emission. If females are attracted, mating occurs on the host plant, and then mated females seek plants for oviposition (Uhler 1951). Male *E. solidaginis* can release more than 70 μg of *E*,*S*‐conophthorin over 24 h (Helms et al. 2013). The volatile emission from male *E. solidaginis* primes *S. altissima* against galling by female *E. solidaginis* (Yip et al. 2021), as well as co‐occurring members of the community of herbivores that feed upon *S. altissima* (Helms et al. 2013; Yip et al. 2017, 2025). Furthermore, some of this evidence suggests that priming is mediated at least in part by JA (Helms et al. 2013). However, transcriptional aspects of this enhanced defence have remained unexplored. This host‐parasite interaction, already a classic ecology system (Abrahamson and Weis 1997), presents a promising model to study priming induced by an insect‐derived volatile cue.

The sensitivity of *S. altissima* to olfactory cues from *E. solidaginis* has presumably emerged as an indicator of future damage from their gall‐inducing larvae, which are major resource sinks for attacked plants (Abrahamson and Weis 1997). Priming appears to convey tolerance of galling, as galled plants with prior *E. solidaginis* cue exposure had significantly higher floral mass and stem height than unexposed plants (Yip et al. 2025). This evidence of priming‐induced compensatory growth when galled, plus heightened defence when damaged by co‐occurring herbivores, illustrates adaptive benefits of priming against insects with a co‐evolutionary history with *S. altissima*.

Importantly, however, no study to date has examined genome‐wide changes in gene expression when plants are primed by insect‐derived volatile cues. Moreover, few studies of plant transcriptomes have focused on generalist herbivores feeding on atypical host plant species (e.g., Bui et al. 2018), presumably a common occurrence in nature. Because co‐evolved herbivores may have adapted to specific defensive responses, insight from non‐co‐evolved interactions would be helpful to understand the baseline primed defence response and the general advantages it may convey. Furthermore, because VOC cues can prime some defence traits while directly inducing others (Kim and Felton 2013), analysing the transcriptomic responses of a plant species to a specific volatile cue with and without subsequent herbivory provides an opportunity to understand which genes may be involved in priming (i.e., preparing for attack) and those involved in defending against attack.

Leveraging our previous findings on volatile‐mediated defence in this ecological model system, we studied how a priming cue from a co‐evolved herbivore affects plant defences generally. Our experiment integrated whole‐transcriptome sequencing with measurements of defence hormones (JA, SA, ABA) to track primed defence in *S. altissima* over 2 days. We designed a time series of 48 h to characterise transcriptomic dynamics of priming to a cue from a specialist herbivore and then defence induction against generalist herbivory. We hypothesised that (i) exposure to a priming stimulus from a co‐evolved herbivore quickens and/or intensifies defence responses to a non‐co‐evolved herbivore across 48 h, and (ii) primed plants increase expression of genes underlying defence signalling and regulation, JA synthesis and downstream defences such as metabolite synthesis, during herbivory from the non‐co‐evolved herbivore. We used corn earworm (*Helicoverpa zea*, Lepidoptera: Noctuidae), a widespread agricultural pest, in our feeding assays because it has no known co‐evolved relationship with *S. altissima* and is broadly polyphagous (Fitt 1989). Our study comprised a factorial design of priming (primed/naïve) and herbivory (damaged/undamaged) to isolate the effects of these conditions and their interaction.

## 2 Materials and Methods

### 2.1 Cultivation of *S. altissima* and *E. solidaginis*

We collected rhizomes of a single genotype of *S. altissima* from an old field near State College, PA (collection site ‘S110’) and propagated rhizomes over several generations in the greenhouse. Before the experiment, we stored rhizomes at 4°C for a minimum of 60 days to simulate vernalisation, then cut rhizomes into ~5 cm segments. We planted rhizome segments in Pro‐Mix BX general‐purpose growing medium with mycorrhizae (Premier Horticulture Inc., Quakertown, PA, USA) on March 16, 2023. After new sprouts grew from rhizomes (~3–5 weeks), we transplanted them into 6‐inch pots in a pest‐free greenhouse. We grew the plants under natural sunlight during the months of March–April 2023, and used 15–25 cm‐tall plants for assays.

### 2.2 Collection of Volatile Emissions From *E. solidaginis* Males

To source *E. solidaginis* males, we collected galls on senesced *S. altissima* plants in an old field in Bellefonte, PA, USA, between December 2022 and February 2023 and stored them at −20°C to maintain larval diapause. When adults were needed, we removed the galls from the freezer and incubated them at room temperature to induce pupation and emergence. Adults are easily sexed by the presence of the female's ovipositor.

Following Helms et al. (2013), we collected volatile emissions (i.e., putative sex pheromones) from male *E. solidaginis* by aerating adult male flies in glass chambers with Teflon or aluminium bases. We pushed filtered house air into the chambers at 0.6 L · min^−1^ and pulled air out of the chambers through filters packed with 45 mg of Super‐Q adsorbent (Alltech Associates, Deerfield, IL, USA) at 0.5 L · min^−1^. We eluted filters with 150 μL of dichloromethane and pooled eluate from each filter to ensure a uniform concentration of emission for the exposure treatments.

### 2.3 Experimental Exposure to *E. solidaginis* Pheromone and *H. zea* Feeding Assay

To examine interactive effects of herbivore damage and defence priming, we assigned *S. altissima* plants to one of four treatment groups: (i) naïve (never exposed) to the priming cue and not damaged by *H. zea*, (ii) primed (exposed to the priming cue) and not damaged by *H. zea*, (iii) naïve and damaged by *H. zea*, or (iv) primed and damaged by *H. zea* (Figure S1). We then introduced *H*. *zea* caterpillars and allowed them to feed for 48 h. We randomly assigned independent plants to 0, 3, 6, 24 or 48 h harvest time points. Each time point had five plants for a total of 100 biological replicates.

For the priming treatment, we exposed plants to a ~50% dose of *E. solidaginis* male emission over a 3‐day period (~70 µg per day at full strength; in our experiment, we used 35 µg per day). We chose this concentration because exposure to a 50% dose of *E. solidaginis* emission leads to the lowest gall formation rate in the experimental *S. altissima* genotype (‘S110’) (E. Yip, unpublished data). In the greenhouse, we attached 2 mL vials filled with 112 μg of male *E. solidaginis* emission diluted in 1.5 mL dichloromethane to 30 cm bamboo stakes and placed one in each pot such that each vial was next to the apical bud of the plant. Each vial had a 4 cm cotton string wick threaded through the rubber insert of the cap to disperse the contents by evaporation. This vial position mimicked the tendency of male *E. solidaginis* to climb to the highest point on *S. altissima* when calling for mates (Uhler 1951). Plants in naïve treatments were exposed to vials of dichloromethane only. The wicked vials remained next to the buds for 3 d.

Immediately after removing the vials, we harvested two 100 mg portions of undamaged leaf tissue from each plant of the 0 h treatment group. These leaves were selected at random from the oldest 20 leaves. We immediately froze both portions in liquid nitrogen and stored them at −80°C for later phytohormone quantification and RNA extraction, respectively. We then placed two fourth‐instar *H. zea* caterpillars on each plant and a mesh bag over each pot to contain the caterpillars. After introducing *H. zea* caterpillars, we followed the same leaf harvesting protocol to take damaged leaf tissue at 3, 6, 24 and 48 hpi (hours post‐infestation).

### 2.4 Analysis of the *S. altissima* Transcriptome

We extracted total RNA from leaves of *S. altissima*, with the Spectrum Plant Total RNA Kit (Sigma‐Aldrich, St. Louis, MO, USA) and followed the standard protocol. We selected RNA extracts from 0, 6, 24 and 48 hpi time point samples based on their RNA integrity number (RIN > 6.0). Novogene (Sacramento, CA, USA) performed paired‐end 150 bp Illumina NovaSeq generating > 50 million reads per library. We trimmed reads using Trimmomatic (options: ILLUMINACLIP:TruSeq. 3‐PE.fa:2:30:10 SLIDINGWINDOW:6:5 LEADING:20 TRAILING:10 MINLEN:30) and pooled the reads from all libraries to assemble the transcriptome de novo with the Trinity pipeline (v2.15.1; options selected when running Trinity: min_contig_length 200, min_kmer_cov 2, no_parallel_norm_stats; Haas et al. 2013). To estimate transcript abundance (gene and isoform levels), we used Salmon v1.4.0 within Trinity and then used BUSCO v5.5.0 to estimate transcriptome completeness (Manni et al. 2021). We predicted coding regions of transcripts with TransDecoder v5.5.0 (Haas n.d.) and searched predicted open reading frames (ORFs) with blastp v2.2.31 and hmmsearch v3.1b.2 against BLAST and Pfam databases, respectively, using default settings. We annotated the predicted peptides with eggNOG‐mapper v5.0 (Cantalapiedra et al. 2021; Huerta‐cepas et al. 2019), limiting the taxonomic scope to only retrieve annotations by homology to the Viridiplantae at maximum taxonomic level and the asterids at minimum taxonomic level. We built the eggNOG‐mapper annotations into an annotation database with R packages AnnotationDBI v1.64.1 and AnnotationForge v1.44.0 (Pagès et al. 2023; Carlson and Pagès 2024). Conclusions we draw about the biological relevance of our findings are limited by incomplete annotation information. Only half of all DEGs could be functionally annotated; GO enrichment of this transcriptome may therefore be biased toward well‐characterised genes in canonical pathways. Nevertheless, defence‐related functional categories dominated DEG profiles (Table S5), and our GO enrichment results agree with the findings of previous studies on plant transcriptome profiles during lepidopteran pest feeding (Garcia et al. 2021; Schweizer et al. 2013). All scripts and outputs for our transcriptome assembly and downstream analyses are publicly available through the Penn State ScholarSphere repository using the digital object identifier doi:10.26207/x31p‐m705.

We analysed gene count data for differential expression with DESeq2 v1.44.0 using default settings (Love et al. 2014). The significance thresholds for differentially expressed genes were log_2_(fold‐change) > 2 and Benjamini‐Hochberg adjusted *p* < 0.05. We normalised count data by library size and variance‐stabilising transformation according to default options in DESeq2; only transformed count data are listed, unless otherwise indicated. We visualised differentially expressed gene quantities with the ggVennDiagram v1.5.2 R package (Gao et al. 2024) and performed gene ontology (GO) enrichment analysis with clusterProfiler v4.10.1 (Xu et al. 2024). We performed weighted gene correlation network analysis (Langfelder and Horvath 2008) on our transcriptome but did not report it as no meaningful patterns emerged; no group of genes strongly correlated with an experimental condition or factorial combination. We instead hierarchically clustered DEGs by their expression profile through time using the DEGreport R package (v1.38.5; options: minimum genes per cluster = 30; Pantano 2017). For analysis of DEGs in defence gene groups, we selected gene family members from our transcriptome based on the presence of functional domains in our PFAM annotations. For a focused examination of expression, we manually curated gene lists for the following families from the literature: JA biosynthesis (Ruan et al. 2019), terpene synthases and prenyltransferases (Priya et al. 2018), flavonoid biosynthesis (Du et al. 2024; Petrussa et al. 2013), pathogenesis‐related (PR) genes (Han and Schneiter 2024), proteinase inhibitors (PIs) (Santamaría et al. 2014), receptor‐like kinases (RLKs) from the metaRLK database (Liu et al. 2024), and defence‐related TFs found on the Plant Transcription Factor Database v5.0 (Jin et al. 2017).

### 2.5 Phytohormone Quantification With GC‐MS

We extracted phytohormones from the other 100 mg portion of each sampled leaf per plant (0, 3, 6, 24 and 48 hpi; *n* = 100 biological replicates). We adapted the vapour phase extraction protocol for collecting leaf hormones from Schmelz et al. (2004). Briefly, we homogenised frozen leaf tissue in 400 μL of extraction buffer then added 1 mL dichloromethane and centrifuged the homogenised tissue. We dried the nonpolar fraction of the extract supernatant then added trimethylsilyldiazomethane to methylate the phytohormones. We volatilised each sample by heating and collected the vapour phase by vacuum filtration using filters packed with 45 mg of Super‐Q adsorbent, then eluted the filters into vials with 150 mL dichloromethane for gas chromatography‐coupled mass spectrometry in positive CI mode using isobutane and select ion monitoring. We quantified JA, SA and ABA by comparing their abundance to that of internal standards (methyl dihydrojasmonate, ^2^H_6_‐SA, ^2^H_6_‐ABA).

### 2.6 Statistics

**Figure 1:**
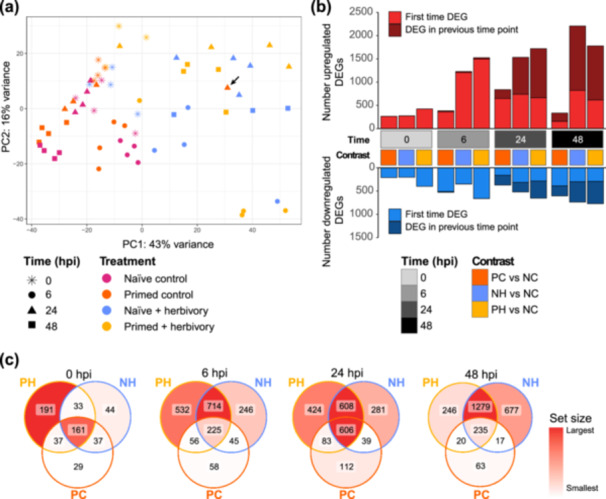
Transcriptomic analysis of naïve and primed defence response in *S. altissima*. (a) Principal component analysis of variance‐stabilised normalised counts data shows herbivory contributes most to sample separation. Normalisation was performed with variance‐stabilising transformation in the DESeq2 software package. Principal component 1, which accounts for 43% of sample variance, appears to separate samples by herbivory. Principal component 2, 16% of the variance, separates samples generally by time. Black arrow indicates primed control replicate with unexpected transcriptomic profile referenced in the text. Each treatment group comprised *n* = 4 biological replicates per time point, except primed herbivory plants at 0 hpi and naïve herbivory plants at 48 hpi, where *n* = 3. (b) Total number of differentially expressed genes (DEGs) at each time point when contrasting treatment groups with the naïve control (NC) group; bars represent the number of genes significantly up‐ or downregulated in treated plants compared to control (naïve, undamaged) plants at each time. Counts of DEGs at 0 h represent the effect of priming alone, while at 6–24 h *H. zea* larvae were feeding. Herbivory appeared to increase gene expression to a greater degree in primed damaged plants than naïve damaged plants at first, while at 48 h, naïve damaged plants had more genes upregulated. Genes with Benjamini–Hochberg adjusted *p* < 0.05 and |log_2_‐fold change| > 2 were considered significant. (c) Venn diagrams of shared sets of DEGs between treatment contrasts at each time point. hpi, hours post infestation; NC, naïve control; NH, naïve with herbivory; PC, primed control; PH, primed with herbivory.

We used R version 4.3.2 (R Core Team 2023) for all statistical analyses. To identify differences in expression profile clusters from DEGreport, we modelled the *Z*‐scores of normalised count data as a three‐way interaction: gene *Z* score of abundance ~ priming × herbivory × time. We performed ANOVA on each cluster followed by Tukey's honestly significant differences (HSD) test and considered differences between means within a time point significant at *p* < 0.001. We applied these statistical tests to all clusters. We used ChatGPT (v4o, OpenAI) to generate R code for compiling Figure 1 and Table S7, as well as general debugging; code that was generated by ChatGPT is marked where applicable in R scripts available under the Penn State ScholarSphere repository.

Phytohormone data were also tested for interaction of time and exposure treatment (naïve undamaged, primed undamaged, naïve damaged, primed damaged) with a three‐way linear model: phytohormone quantity (ng·g leaf tissue^−1^) ~ exposure × herbivory × time. The residuals of these models for each phytohormone were normally distributed after natural log transformation. We performed ANOVA on each model and considered interaction terms between factors significant when *p* < 0.05. We computed a post hoc Tukey's HSD test to determine whether means were significantly different within a time point.

## 3 Results

### 3.1 Transcriptome Statistics and Differential Expression Analysis

Our de novo assembly of the *S. altissima* transcriptome (*n* = 62 libraries) resulted in 1 201 142 transcripts of 659 519 predicted Trinity genes. The gene‐level contig N50 was 503, with a median contig length of 281 (for more details on sequencing and assembly statistics, see Table S1). Principal component analysis of normalised gene counts suggested that separation of samples was most strongly influenced by herbivory and time (Figure 1a). The *S. altissima* population we sampled is likely hexaploid (6*n* = 54, expected genome size > 5 pg DNA; Yip, unpublished data; Halverson et al. 2008; Etterson et al. 2016). Because our assembly comprised more than 650 000 predicted genes, it is possible that fragmented transcripts inflated the alignment process in Trinity. Many of these predicted genes had fewer than 10 counts after transcript quantification. Overall, 75 378 genes could be annotated from the transcriptome by identity to published genomic resources (Table S2). Because we did not specify the strandedness of our transcriptome in TransDecoder, approximately 30% of our predicted ORFs were in the antisense orientation. Although this may have generated some artificial gene annotations, it is unlikely that this affected our GO enrichment analysis because annotations based on antisense ORFs were not applied to any significant differentially expressed genes.

Within each time point, we used naïve undamaged plants as a baseline for screening differentially expressed genes (DEGs, |log_2_(fold‐change) [LFC]| > 2, adjusted *p* < 0.05). In total, 4213 unique genes were significantly up‐ or downregulated in any treatment comparison (Table S2, Figure 1b). Of all DEGs, 2184 had annotation hits (2.9% of annotated genes, 51.8% of DEGs, Table S2).

### 3.2 Priming Augments Gene Expression for First 24 h of Herbivory

Following 3 day of exposure to the priming cue at 0 hpi, few genes were significantly upregulated in the treatment groups compared to naïve controls (Figure 1b), and about half were not shared between any treatments (Figure 1c). Adding *H. zea* larvae greatly increased DEGs at 6 hpi and upregulated more genes in primed than naïve plants (Figure 1b). The 6 hpi time point occurred at night, so comparisons between expression levels at 6 hpi and other times could include circadian effects. At 24 hpi, primed control plants had large increases in upregulated DEGs, many of which were also present in the damaged treatments (Figure 1c), though this could be due to a single replicate whose overall transcriptome profile resembled damaged plants at that time (Figure 1a, arrow). Primed and naïve damaged plants shared the largest sets of upregulated genes during herbivory (Figure 1c; for shared downregulated gene sets, see Figure S2). Herbivory induced more genes exclusive to primed plants at 6 and 24 hpi, but by 48 hpi, naïve plants had more unique DEGs. Priming thus augmented overall gene expression for the first 24 h of herbivory, but this effect diminished by 48 hpi.

We expected significant DEGs present in both primed treatments to indicate the transcriptional effect of priming. Few GO terms were significantly enriched in any set of genes shared between primed damaged and primed control plants at any time point (adjusted *p* > 0.05; Table S7); nevertheless, many of the annotated DEGs in these sets were related to defence, despite the primed control plants not receiving herbivory (Table S6). At 0 hpi when herbivores were absent, both primed treatments shared only 37 upregulated DEGs (Figure 1c), which included at least one putative terpene synthase (Table S6). After 6 h of herbivory, both primed treatments significantly upregulated DEGs putatively related to defence gene regulation and secondary metabolite synthesis (Table S6). At 24 hpi, primed control plants shared 83 upregulated DEGs with primed damaged plants (Figure 1c), including genes involved in antimicrobial defence such as *EDS1* (Dongus and Parker 2021; Table S6). The three‐way shared 24 hpi set was enriched in defence‐related GO terms such as response to JA and anthocyanin biosynthesis, suggesting that both primed treatments did not differ greatly from naïve plants at 24 hpi (Figure 1c, Table S7). By 48 hpi, there were only four annotated DEGs shared between primed treatments (Table S6).

### 3.3 Time‐Based Clustering Revealed a Phase Shift in Primed Defence Gene Expression

**Figure 2:**
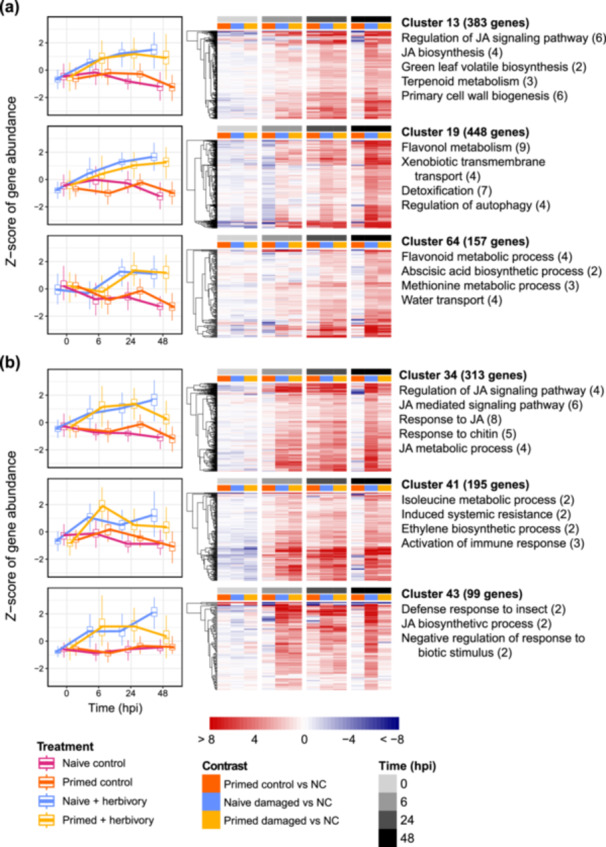
Time‐based profiling of DEGs revealed two major defence expression patterns. Genes significantly upregulated at any time during herbivory (*n* = 2650) were sorted into 22 groups using unsupervised hierarchical clustering. Line graphs display *Z*‐score of abundance of normalised gene counts. Heatmaps display log_2_‐fold change difference in gene counts for each treatment compared to the naïve undamaged control (NC) at each time point. Selected enriched gene ontology terms for each cluster are listed with the number of genes contributing to each term's enrichment (Benjamini–Hochberg‐adjusted *p* value < 0.05). (a) Selected clusters representative of conventional defence response. DEGs in the conventional pattern increased in their expression in damaged plants over 48 h of feeding. (b) Selected clusters representative of priming‐mediated phase shift. On average, genes in the phase shift pattern were more abundant at 6 h but less abundant at 48 h in primed damaged plants. Hpi, hours post infestation.

We used hierarchical clustering to identify temporal patterns in gene expression due to priming and herbivory. Using the set of all significant upregulated DEGs from all time points, the cluster analysis grouped 2650 genes into 22 clusters based on normalised abundance across time (Figure 2 and Figure S3). We performed GO enrichment analysis for each cluster of genes to identify biological themes in shared expression profiles (Figure 2, Table S5). We selected clusters whose expression profile and functional enrichment were relevant to our hypotheses.

Two major patterns emerged across the clusters for the 48‐h feeding period: (i) a progressive increase in the abundance of genes expressed in primed and naïve damaged plants, that, a ‘standard’ phase of intensifying defences as herbivory continued (Figure 2a); and (ii) an increase in abundance until 24 hpi, but a decrease from 24–48 hpi, that, a shortened or ‘advanced’ defence phase that peaked before 48 hpi (Figure 2b). Clusters 13, 19 and 64 were representative of the standard phase (a combined total of 988 genes, 37.3% of all clustered DEGs); on average those genes increased in primed and naïve damaged plants by approximately 1–1.5 standard deviations (SD) over the feeding period, but did not increase in the two undamaged treatments (Figure 2a). Genes in standard phase clusters were most significantly enriched in defence‐related functional categories such as flavonoid metabolic process (GO:0009812), detoxification (GO:0098754), sesquiterpenoid biosynthetic process (GO:0016106) and JA biosynthesis (GO:0009695; Figure 2a, Table S4). In cluster 13, for example, one *LOX* and one *ILL6*, plus two thiolase‐like genes, contributed to significant enrichment of JA biosynthesis GO terms (Table S5). Cluster 13 also contained four TIFY and CCT_2 domain‐containing genes that contributed to enrichment of JA signalling pathway regulation (GO:2000022); these domains are conserved in the JAZ family of JA synthesis repressors (Zhang et al. 2020; Table S5). Clusters 13, 19 and 64 contained multiple homologues of the flavonoid biosynthesis pathway genes *PAL*, *CHS* and *PPO*, plus more than 50 genes annotated as belonging to the UDP‐glycosyltransferase family, which features in anthocyanin and other defence flavonoid biosynthesis (Ren et al. 2025; Figure S6d, Table S4). Four putative terpene synthases were also present across the standard phase clusters, which contributed to some GO enrichment for terpenoid biosynthesis (Table S5).

In primed damaged plants, some defence‐related genes appeared to follow an advanced phase pattern (Figure 2b). Genes in advanced phase clusters 34, 41 and 43 (total 607 genes, 22.9% of all clustered DEGs; Table S4) were approximately 1–1.5 SD less abundant on average in primed damaged plants than in naïve damaged plants at 48 h (Figure 2b). The 313 genes in cluster 34 were highly enriched in defence GO terms and included key JA biosynthesis genes *AOS*, *AOC* and *ACX*, plus methyl‐JA conjugator *JMT* (Tables S4, S5). At 6 hpi, genes in cluster 41 were on average more abundant by 0.83 of the SD in primed plants than naïve plants, indicating that they are more highly induced during primed defence (Figure 2b). Overall, genes in the advanced phase clusters were most significantly enriched in defence‐related functional categories such as JA biosynthetic process (GO:0009695), response to JA (GO:0009753), JA mediated signalling pathway (GO:0009864), defence response to insect (GO:0002213) and regulation of response to biotic stimulus (GO:0002831; Figure 2b, Table S4). Clusters 34, 41 and 43 were not significantly enriched in terpenoid or flavonoid biosynthesis but did contain a combined total of seven putative terpene synthases (Table S8).

### 3.4 Priming Intensified Defence‐Related Gene Expression During Herbivory

**Figure 3:**
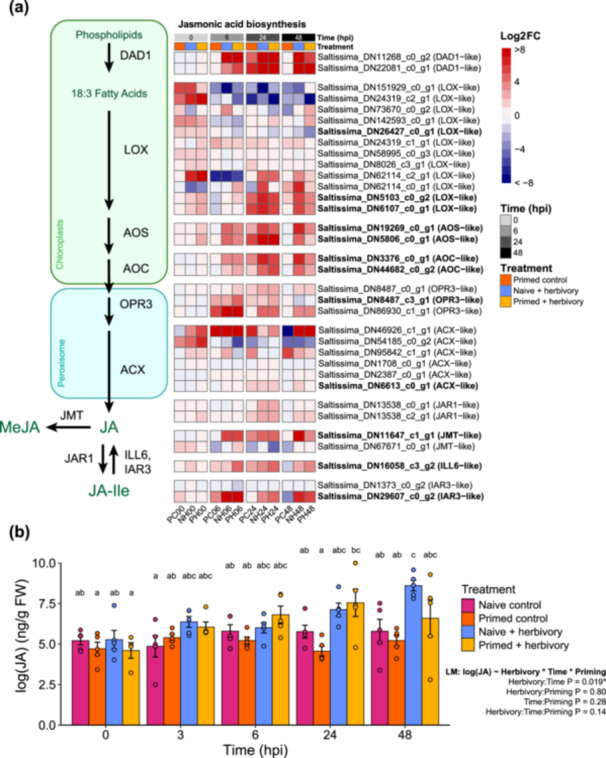
Priming differentially affected JA biosynthesis gene expression. (a) Schematic of canonical JA biosynthesis pathway and heatmap of gene expression. Pathway metabolites are in green. Heatmap of log_2_‐fold change differential expression of genes in the JA biosynthesis pathway. Gene names in bold are significant DEGs (FDR < 0.05, |log_2_(fold change)| > 2. (b) Leaf JA content. Each treatment group comprised *n* = 5 biological replicates per time point, except primed herbivory plants at 0 hpi, where *n* = 4. There were no significant differences between primed and naïve herbivory at 48 h. Different letters represent significant differences between treatment means across all time points. Error bars represent mean total (±SE) ng · g^−1^ fresh tissue leaf phytohormones produced throughout 48 h of *H. zea* feeding on *S. altissima*. Data shown are log‐transformed. Linear model significance level of *p < 0.05 (Tukey HSD). Hpi, hours post infestation.

We identified signatures of primed expression in well‐known defence pathways, including defence‐related TFs and RLKs (Figure S4), JA biosynthesis (Figure 3), PR and PI genes (Figure S5) and terpenoid and flavonoid biosynthesis (Figure S6).

#### 3.4.1 TFs and RLKs

Priming theoretically intensifies plant defence early in an herbivore attack. We therefore expected to identify TFs upstream of defence that were more abundant in primed damaged plants. At 6 hpi, the closest time point to the start of herbivory, homologues of the ethylene‐sensitive AP2 superfamily, auxin‐sensitive B3 superfamily and the stress‐related MYB and NAC superfamilies were highly expressed in primed damaged plants (Figure S4a, Table S3). These TF families have been extensively studied for their role in defence response (Feng et al. 2020; Goossens et al. 2017; Nuruzzaman et al. 2013).

Both primed and naïve damaged treatments showed significant up‐regulation of most of the TFs in our curated set at 6, 24 and 48 hpi, but differed in their magnitude of expression (Figure S4a). By 48 hpi, multiple *AP2*, *MYB* and *NAC* TFs had lower LFC in primed damaged plants (Figure S4a). Notably, primed control plants showed significant upregulation of *WRKY*, *AP2* and *MYB* TF genes variously at 6, 24 and 48 hpi despite being undamaged (Table S4).

Besides TFs, we examined expression profiles of RLK involved in defence. We observed a differential effect of priming on one thaumatin‐like (*PR‐5*‐like) protein kinase (PF00314), which is involved in antifungal defence (Han and Schneiter 2024), and one GDPD domain‐containing protein (PF03009), a group whose members are expressed following pathogen infestation (Liu et al. 2025; Figure S4b). Primed damaged plants showed relatively low expression of another GDPD domain‐containing gene, which was highly upregulated at 6 and 24 hpi but low at 48 hpi (Figure S4a).

#### 3.4.2 JA Biosynthesis Genes

During *H. zea* feeding, we hypothesised that primed plants would more strongly express genes related to JA biosynthesis based on previous findings (Helms et al. 2013). At 6 hpi, primed damaged plants did not have higher induction of JA biosynthesis genes than naïve damaged plants; major JA biosynthesis reporter genes *LOX*, *AOS* and *AOC*, as well as JA‐activating *JAR1*, showed varying intensity and significance of expression at this time (Figure 3a). By 24 hpi, these genes showed higher levels of induction in primed and naïve damaged plants. Notably, wound response reporter genes *LOX*, *AOS* and *AOC* were significantly upregulated in undamaged primed plants at 24 h (adjusted *p* < 0.05; Figure 3a), suggesting that these genes are responsive to priming and can be induced in a primed state without herbivore damage. By 48 hpi, *LOX*, *AOS*, *AOC*, *ACX*, MeJA conjugator *JMT*, and JA‐Ile inactivator *IAR3* were more highly expressed in naïve damaged plants than primed damaged plants (Figure 3a). While these genes were nonetheless significantly upregulated in primed damaged plants at 48 hpi (Table S3), their relatively lower expression indicates decreased JA activity late in the primed defence response. Moreover, this pattern in JA biosynthesis gene expression agrees with the overall trend we observed in our hierarchical clustering of upregulated DEGs, where JA biosynthesis and regulation gene categories displayed a shorter phase of expression on average in primed plants (Figure 2b).

#### 3.4.3 PR and PI Genes

The PR and PI gene groups are important members of biotic stress response in plants, some of which have been studied in the context of priming (Mauch‐Mani et al. 2017). There were 39 significant DEGs annotated as PR genes. Many PR DEGs showed higher induction in primed plants than in naïve damaged plants from 6 to 24 hpi (Figure S5a). Notably, multiple *PR‐3*‐ and *PR‐8*‐like chitinases were highly induced (4.1‐9.1 LFC vs naïve control) at 6 hpi and substantially higher in primed damaged plants than in naïve damaged plants (Table S3). Many of the early‐induced PR genes in primed damaged plants increased or maintained expression levels at 24 and 48 hpi, during which naïve damaged plants also showed induction (Figure S5a, Table S3). Though not canonical PR proteins, multiple RNase T2 domain‐containing genes were significantly upregulated in primed damaged plants at 6 hpi, remained upregulated throughout the feeding assay, and were highly expressed in primed control plants at 24 hpi (Figure S5, Table S3); T2 ribonucleases have been implicated in defence against both pathogens and insects (MacIntosh 2011). Two β‐1,3‐glucanase (*PR‐2*‐like) and one peroxidase 10 (*PR‐9*‐like) gene were significantly upregulated in primed control plants at 6 hpi (Table S3); PR‐2 is known to degrade microbial cell walls and co‐expresses with chitinase PR proteins (Han and Schneiter 2024).

There were only 10 significant DEGs with canonical PI domains expressed during the experiment (Figure S5b, Table S3). Most showed higher expression in naïve damaged plants in comparison to primed damaged plants (Figure S5b). Three Kunitz_legume domain‐containing PIs, which are known to inhibit hydrolysing cysteine and serine proteinases of herbivorous insects (Oliva et al. 2010), were induced in primed damaged plants at 0 hpi and remained upregulated through 48 hpi, but were less abundant in primed plants than naïve plants during herbivory (Figure S5b, Table S3).

#### 3.4.4 Terpenoid and Flavonoid Biosynthesis Genes

We also identified DEGs involved in terpenoid and flavonoid compound biosynthesis, which are major defensive resources against herbivores. *Solidago altissima* has a diverse and well‐studied profile of terpenoid and flavonoid compounds (Heath et al. 2014); essential oils from aerial plant portions have demonstrated antifungal activity (Lawson et al. 2020). Going by predicted functional domains, we detected 18 significant prenyltransferase‐ and terpene synthase‐like DEGs, which carry out the major early steps of general terpene biosynthesis (Figure S6c). Due to the huge number of specialised terpenoid compounds present in plants and the variation in their metabolic pathways, we focused on these general enzymes to capture gross terpenoid induction. Overall, the putative prenyltransferases and terpene synthases were highly expressed in the two damaged treatments with subtle differences in expression between primed and naïve plants (Figure S6a). There was no strong pattern of terpene synthase or prenyltransferase expression in primed plants (Figure S6a). Compared to naïve damaged plants, many terpene synthases were substantially less induced in primed damaged plants at 48 hpi (Figure S6a).

We similarly identified 39 significant DEGs involved in flavonoid biosynthesis using predicted functional domains (Figure S6d). Some homologues of key genes early in this pathway, including chalcone synthase (*CHS*), chalcone isomerase (*CHI*), as well as flavanone‐producing *F3'H* and anthocyanidin‐producing *DFR* and *ANS*, were induced at 6 hpi in primed damaged plants only (Figure S6d, Table S3). These genes were upregulated in both damaged treatments at 24 and 48 hpi, to a relatively higher degree in primed plants (Figure S6d, Table S3).

### 3.5 Phytohormone Quantification With GC‐MS and *H. zea* Feeding Assay

**Figure 4:**
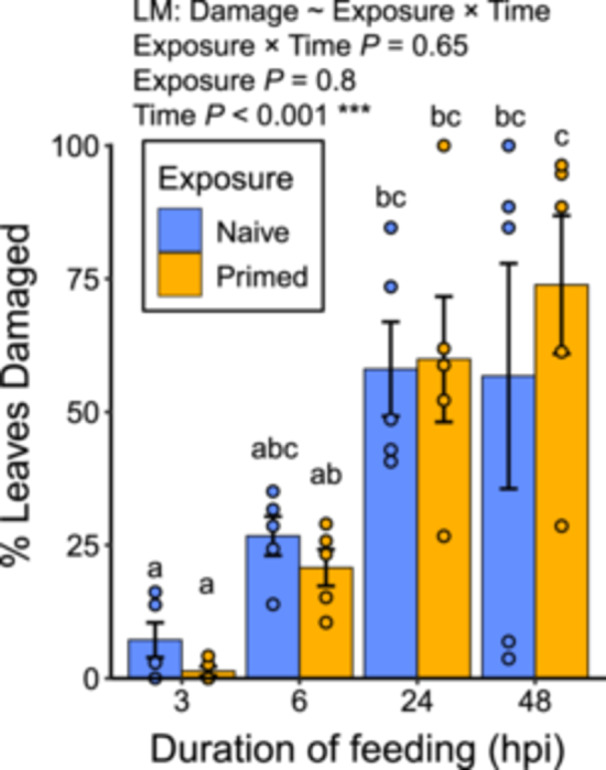
*Helicoverpa zea* damage on *S. altissima* plants increased with time, but was not affected by *E. solidaginis* exposure. Proportional damage was calculated as the ratio of leaves with any damage to intact leaves per plant. Different letters indicate statistical differences between treatment means across all time points (*p* < 0.05, Tukey's HSD test). Effects of exposure and time based on ANOVA (*F*_3,32_ = 0.54, *p* = 0.65). Error bars indicate 1 standard error of the mean (*n* = 5). Hpi, hours post infestation.

Despite a trend of differential effects of priming on defence DEG abundance, there were no significant differences of total JA levels between primed and naïve damaged plants within any time point (Figure 3b; Tukey HSD *p* > 0.05). Considering only primed damaged plants, JA levels were highest at 24 hpi and significantly higher than at 0 hpi (*p* = 0.028), but levels at 24–48 hpi were similar (*p* = 0.99) (Tukey's HSD tests). In naïve damaged plants, JA peaked at 48 hpi and was significantly higher than at 0 h (*p* = 0.0021). Earlier induction of JA after priming agrees with previous research (Helms et al. 2013; Engelberth et al. 2007), but a decrease in JA after 24 hpi may reflect the phase shift in defence gene expression that we observed in the transcriptomes of primed plants (Figure 2b). The observed rise and fall of JA over 48 h in primed damaged plants suggests accelerated defence induction, but a return to a lower defensive state. In addition, the respective JA peaks at 24 hpi in primed plants and 48 hpi in naïve plants may help explain the mild but opposite trend of *H. zea* damage peaking at 48 hpi in primed plants and 24 hpi in naïve plants. Uncontrolled variation in our JA data is likely attributable to the variability of insect feeding, as the *H. zea* caterpillars moved widely across the body of the plant and did not appear to feed continuously. Neither priming nor caterpillar damage significantly affected levels of SA or ABA (Figure S7). The proportion of leaves damaged by *H. zea* did not significantly differ between treatments within any time point, while damage increased significantly from 6 to 48 hpi within primed plants but not within naïve plants (Figure 4).

## 4 Discussion

In this study, to understand how a priming cue from a co‐evolved herbivore affects plant defences generally, we primed defences of *S. altissima* by exposing plants to volatile emissions of *E. solidaginis* males before 48 h of feeding from *H. zea* caterpillars. We then measured transcriptional and phytohormone responses of *S. altissima* to herbivory with and without primed defence. Overall, we found that prior exposure to a specific threat cue changed expression patterns of hundreds of defence‐related genes during herbivory. These modifications amounted to a defensive phase shift in primed plants. A set of defence‐related TFs, RLKs, PR and anthocyanin biosynthesis genes appeared to be primed by exposure to the *E. solidaginis* emission, which confirms that the volatile cue of a specialist herbivore can prime plant defences against an unrelated generalist. Notably, priming did not appear to deter generalist feeding in this study despite the transcriptional phase shift, likely because the generalist herbivore was not sensitive to the defences deployed by *S. altissima*.

### 4.1 Priming Intensified Expression of Anti‐Herbivore and Immune Response Genes

Our differential expression analysis identified a set of defence‐related genes that appeared to respond to the priming cue from *E. solidaginis*. The overall transcriptional signature of primed defence included JA‐ and ET‐sensitive TFs, antifungal RLKs, chitinases and anthocyanin biosynthesis genes. To date, this is the only study to report a concurrent primed response of each of these pathways. Genes annotated with terpene biosynthesis‐ and PI‐related functional domains showed little evidence of primed expression, though our focused analysis of these gene groups was limited by our de novo transcriptome annotation.

Primed response to herbivory is hypothesised to involve signalling molecules including JA and ET and to be mediated first by TFs associated with those hormones (Mauch‐Mani et al. 2017). Previous research suggested that priming potentiates defence by stockpiling defence TFs such as *MYC2*, leading to higher expression during attack of downstream defences (Vos et al. 2013). In our experiment, we observed little evidence of TF stockpiling at 0 hpi, but found multiple ethylene‐sensitive *AP2* and JA‐related *NAC* TFs highly upregulated upon *H. zea* infestation (Figure S4a). The increase of the former TF also suggests that primed plants may be more sensitive to ET signalling from neighbouring plants.

This group of TFs was not significantly upregulated at 0 hpi (Table S3), so we cannot conclude that they were abundant in the absence of herbivores, but their heightened upregulation in primed damaged plants at 6 hpi suggests that the *E. solidaginis* cue primes their expression during herbivory.

Another feature of primed defence was enhanced signal transduction. We profiled defence‐related RLK and identified thaumatin and GDPD domain‐containing RLKs that responded to priming. GDPD family proteins have been linked to diverse functions in plants and recently shown to be expressed during fungal infection (Liu et al. 2025). Thaumatin‐like proteins (canonically *PR‐5*) and antimicrobial defence protein *PR‐1* were upregulated at 6 hpi in primed damaged plants. *PR‐1* has been implicated in recognition of pathogen effectors (Breen et al. 2017), and *PR‐5* is induced during fungal infection (Wang et al. 2010); both are indicators of SA‐mediated defence but may be participating in effector recognition during *H. zea* infestation. These results suggest that *S. altissima* may have enhanced signalling capability in the primed state.

PIs are a well‐characterised means of defence against herbivores, and previous research in tomato (*Solanum lycopersicum*) found that oviposition by *H. zea* strongly primed expression of PI *PIN2* (Kim et al. 2012). However, results suggest that priming did not strongly affect expression of the few DEGs with PI domains during *H. zea* feeding. Therefore, although plants in certain interactions may sensitively prime defences based on specific molecular cues (Kessler and Mueller 2024), PIs may not be an important asset for defences of primed *S. altissima*; alternatively, PI genes could be primed for rapid induction only after detecting molecular patterns associated with feeding *E. solidaginis* larvae.

During herbivory, heightened levels of JA can persist across 48 h (Stroud et al. 2022); therefore, we expected *S. altissima* plants to elevate JA levels throughout our feeding assay. In our experiment, however, JA seemed to have different dynamics in primed and naïve plants. When comparing only within treatment groups, levels of JA were significantly higher than baseline (i.e., pre‐herbivory) at 24 hpi in primed damaged plants and 48 hpi in naïve damaged plants, but due in part to large variation, JA levels did not significantly vary between primed and naïve plants at any point. Despite a lack of significant differences in JA in leaves between primed and naïve plants, our phytohormone results appear to somewhat mirror the results of our transcriptome analyses, as JA levels and JA biosynthesis genes seemed to rise and fall in a similar temporal phase. This apparent phase shift in gene expression has not yet been shown in previous studies on primed defence in plants. A recent study with rice showed that exposure to the volatile chemical *Z*‐3‐hexenyl acetate, without herbivory, triggered wide‐scale transcriptome rearrangements 17 h after elicitation, plus a rise and fall of JA over 41 h (Desmedt et al. 2023) In our analysis, multiple clusters with both standard and advanced phase patterns contained JA biosynthesis, signalling, and regulation genes, suggesting that certain parts of the JA biosynthetic and regulatory pathway were differentially regulated during the primed defence response (Table S3).

Unexpectedly, many defence genes in undamaged primed plants were significantly upregulated at 24 hpi. We calculated LFC of each gene by averaging expression across multiple samples in a treatment, so it is possible that a single replicate is responsible for this uptick in expression, as captured by the separation of a 24 hpi primed control sample in our principal component analysis (Figure 1a). This sample was closely associated with samples from damaged treatments, though the leaf tissue was intact at the time of harvest. An alternative explanation for this result is that primed plants, without receiving damage, were more sensitive to the volatiles of damaged neighbours and so deployed a defence reaction. Defence induction resulting from airborne, herbivore‐associated volatiles alone has been demonstrated (Desmedt et al. 2023), but because we did not see similar induction in primed control plants at other time points, this likely did not occur in our experiment.

### 4.2 Priming Does Not Affect Transcript Accumulation After 3 Days of Continuous Exposure

Primed defence is thought to be mediated in part by the accumulation of defence‐related mRNAs (Hilker et al. 2016). We therefore expected to identify genes with high abundance in primed plants at 0 hpi, the time point immediately after we removed the priming cue, which was present for the three‐day priming phase. At 0 hpi, in the absence of herbivores, a single terpene synthase‐like gene drove GO enrichment of terpenoid biosynthesis in both primed treatment groups (Tables S6, S7). Across TFs, RLKs, JA biosynthesis and secondary metabolite biosynthesis, few genes appeared responsive to the priming stimulus without herbivory (Figures 1b, S3, 3b, S6). Because we sampled this time point 3 days after initial cue exposure, it is possible that our sampling did not capture the hypothesised transcriptional spike at the onset of priming (Martinez‐Medina et al. 2016). This result builds on the current understanding of how plants maintain the primed state: after 3 days of cue exposure, even in the continuous presence of the priming stimulus, the transcriptome of primed plants was indistinguishable from that of naïve plants (Figure 1a). Therefore, preparatory transcription resulting from exposure to the priming stimulus may occur over fewer than 72 h. Another important consideration is whether the hypothetical stockpiled genes persist as mRNA or proteins; protein abundance data are needed to further integrate the importance of translation in the primed state.

### 4.3 Primed Defence Has a Shorter Phase Than Naïve Defence

Aside from details on the primed state, our most striking result was the decline at 48 hpi in large numbers of defence‐related gene expression, especially JA biosynthesis‐related genes. This result suggests that enhanced defences from priming likely last for fewer than 2 days. Studies that examined primed defence for 24 h or longer have not reported such a JA‐related phase shift exclusive to primed plants (Karssemeijer et al. 2022; Coolen et al. 2016; Vos et al. 2013). In our experiment, primed plants that received herbivory showed a clear rise and fall of JA‐related gene expression within a feeding period of 48 h (Figure 2b).

Once herbivores were present at 6 hpi, gene functions related to immune response, JA biosynthesis, ethylene biosynthesis and biotic stress were significantly enriched in gene clusters with higher induction in primed plants (Figure 2b). Our results do not capture immediate plant responses to the priming cue, but we observed in primed plants a stronger induction of defence‐related genes during herbivory. Compared to naïve plants, we cannot conclude that primed defences in *S. altissima* were deployed earlier, but higher average abundances of many defence‐related genes in primed plants at 6 hpi indicate stronger defences in the first 6 h of herbivore attack. Other transcriptome studies of responses to herbivory have shown that, while gene expression may fluctuate based on herbivore species and duration of feeding, plants under attack deploy defences if attackers are present (Montesinos et al. 2024; Tzin et al. 2017). Indeed, in our experiment, both primed and naïve damaged plants significantly upregulated the same defence‐related gene categories. However, our results deviate from the paradigm that defence genes are expressed while attackers are present: when *S. altissima* was exposed to the priming cue and then attacked by *H. zea* caterpillars for 48 h, hundreds of defence‐related genes had returned to near‐baseline expression levels by the end of the feeding period (Figure 2b). This result could reflect decays of primed defence responses before plants return to a basal defensive state. Without data on abundance of defence‐associated proteins, however, it is possible that the reduction in abundance of defence‐related transcripts at 48 hpi does not reflect a lower defensive state in primed plants. Indeed, even large changes in mRNA abundance may have comparatively little effect on protein levels (Schwanhäusser et al. 2011; Liu et al. 2016).

Because priming appears to be a resource‐saving defensive strategy (Kessler and Mueller 2024), plants with prior exposure to a threat should be able to repel attackers with a swift, and possibly stronger, defence response, limiting the cost of long‐term attack. Studies of the short‐term (0–12 h) dynamics of primed defence found that plants exposed to volatiles from damaged neighbours or prior herbivory showed earlier induction of JA‐mediated defences during subsequent attack (Ton et al. 2007; Engelberth et al. 2007). In our study, both standard and advanced phase clusters contained defence genes but differed in key gene categories, which suggests that primed defence does not include all available defensive resources (Figure 2). For example, flavonoid biosynthesis and terpenoid biosynthesis were significantly enriched in clusters with a standard phase and showed higher abundance at 6 hpi in primed plants but increased over 48 h in naïve plants only (Figure 2a). These compounds therefore appear to be important for response to herbivory but may not be ideal for rapidly induced defence because they are expensive and complex to synthesise (Li et al. 2023); induction of costly metabolites may instead be related to long‐term herbivory (Karban 2011). These secondary metabolites may comprise a long‐term defensive programme after initial primed defences decay, similar to how peach trees (*Prunus persica*) deploy extensive terpenoid induction against the aphid *Myzus persicae* 72 h into infestation (Pan et al. 2024).

### 4.4 Implications for Primed Defence Against Generalist Herbivory

In the last decade, insights about interactions between *S. altissima* and *E. solidaginis* have revealed that *S. altissima* is sensitive to the volatile emission of male gall flies (Helms et al. 2013, 2014, 2017). More recent work has shown that *S. altissima* plants primed by *E. solidaginis* are galled at a lower rate by *E. solidaginis* and show increased tolerance to galling, suggesting that priming conveys defence when a threat cue is matched with its associated damage (Yip et al. 2021). In our experiment, we exposed plants to *E. solidaginis* emission and then introduced larvae of *H. zea*, which has no co‐evolutionary relationship with *S. altissima*. In our transcriptomic results for primed undamaged plants, we did not see an obvious pattern that could explain tolerance or higher resistance to galling that could explain our previous results (Figure 1c: PC vs. NC ‘63’ genes; Table S7). Nevertheless, in the non‐co‐evolved scenario we presented here, priming plants with the emission of *E. solidaginis* resulted in intensified gene expression across multiple defence pathways once feeding by *H. zea* began. Our results confirm that damage mismatched with a priming cue can still trigger a primed defence response even if the damage source is unrelated to the threat cue.

Although exposure to the cue from male *E. solidaginis* caused transcriptional changes in *S. altissima*, *H. zea* caterpillars attacked statistically similar proportions of leaves at each time point (Figure 4). *H. zea* feeds on a broad range of plant taxa and lacks a co‐evolved relationship with *S. altissima*, so it may not be slowed by *S. altissima* defences, primed or not, as might a specialist herbivore species. Rather, we expect that the differentially induced defence‐related genes that we observed should be more potent against an ovipositing female *E. solidaginis* or the gall‐inducing larva, both of which can be anticipated following signal of the adult male fly (Helms et al. 2013; Yip et al. 2021). Current results will make a useful comparison in future research on the influence of a specialist priming cue on a specialist herbivore, which we expect to be complex and intertwined due to co‐evolution.

## 5 Conclusions

Here, we present evidence that priming augments the expression of hundreds of genes soon after herbivory begins, including chitin‐degrading enzymes and PR proteins. Furthermore, we show plant defences that are primed following the detection of a specialist's volatile cue can be triggered by damage from an unrelated generalist. Importantly, our results illustrate a priming‐related transcriptomic phase shift on the scale of days, whereas many studies have resolved only the first 6–12 h following the trigger of primed defence against short bouts of herbivory. The current results lead us to hypothesise that any enhanced defences from perceiving an insect‐derived priming cue could last fewer than 2 days. Future molecular studies will be needed to elucidate how long plants take to return to a naïve state after herbivory, and whether repeated exposure to priming cue and damage might engender continually enhanced defence.

## Conflicts of Interest

The authors declare no conflicts of interest.

## Supporting information


**Supplementary Figure S1:** Schematic of experimental design.

**Supplementary Figure S2:** Venn diagrams of downregulated DEGs.

**Supplementary Figure S3:** All time‐based DEG clusters produced in DEGreport.

**Supplementary Figure S4:** Expression of receptor‐like kinase and defense‐related transcription factor genes.

**Supplementary Figure S5.** Expression of pathogenesis‐related and proteinase inhibitor genes.

**Supplementary Figure S6:** Expression of genes in the terpenoid and flavonoid biosynthesis pathways.

**Supplementary Figure S7:** Levels of salicylic acid and abscisic acid.

**Supplementary Table S1:** Transcriptome assembly statistics. **Supplementary Table S2:** Transcriptome annotation hits generated in eggNOGmapper. **Supplementary Table S3:** Differentially expressed genes for all 12 pairwise comparisons. **Supplementary Table S4:** DEGs in all hierarchical clusters. **Supplementary Table S5:** Gene ontology enrichment for all DEG clusters. **Supplementary Table S6:** Venn diagram sets of shared DEGs. **Supplementary Table S7:** Gene ontology enrichment for all shared DEG sets.

Supmat.

## Data Availability

RNA sequencing data for this study are available via the NCBI Sequence Read Archive under BioProject ID PRJNA1295474. Scripts for all analyses, as well as phytohormone quantification and feeding assay data, are available on the Penn State University ScholarSphere repository, https://doi.org/10.26207/x31p-m705.

## References

[pce70279-bib-0001] Abrahamson, W. G. , and A. E. Weis . 1997. Evolutionary Ecology Across Three Trophic Levels: Goldenrods, Gallmakers, and Natural Enemies. Princeton University Press.

[pce70279-bib-0002] Bittner, N. , J. Hundacker , A. Achotegui‐Castells , O. Anderbrant , and M. Hilker . 2019. “Defense of Scots Pine Against Sawfly Eggs (*Diprion pini*) Is Primed by Exposure to Sawfly Sex Pheromones.” Proceedings of the National Academy of Sciences 116, no. 49: 24668–24675. 10.1073/pnas.1910991116. PMC6900732 31748269

[pce70279-bib-0003] Breen, S. , S. J. Williams , M. Outram , B. Kobe , and P. S. Solomon . 2017. “Emerging Insights Into the Functions of Pathogenesis‐Related Protein 1.” Trends in Plant Science 22: 871–879. 28743380 10.1016/j.tplants.2017.06.013

[pce70279-bib-0004] Bui, H. , R. Greenhalgh , A. Ruckert , et al. 2018. “Generalist and Specialist Mite Herbivores Induce Similar Defense Responses in Maize and Barley but Differ in Susceptibility to Benzoxazinoids.” Frontiers in Plant Science 9: 1–19. 30186298 10.3389/fpls.2018.01222 PMC6110934

[pce70279-bib-0005] Cantalapiedra, C. P. , A. Hernández‐Plaza , I. Letunic , P. Bork , and J. Huerta‐Cepas . 2021. “eggNOG‐mapper v2: Functional Annotation, Orthology Assignments, and Domain Prediction at the Metagenomic Scale.” Molecular Biology and Evolution 38: 5825–5829. 34597405 10.1093/molbev/msab293 PMC8662613

[pce70279-bib-0006] Carlson, M. , and H. Pagès 2024. AnnotationForge: Tools for Building SQLite‐Based Annotation Data Packages. R package version 1.44.0. https://bioconductor.org/packages/AnnotationForge.

[pce70279-bib-0008] Coolen, S. , S. Proietti , R. Hickman , et al. 2016. “Transcriptome Dynamics of Arabidopsis During Sequential Biotic and Abiotic Stresses.” Plant Journal 86: 249–267. 10.1111/tpj.13167 26991768

[pce70279-bib-0009] Desmedt, W. , M. Ameye , O. Filipe , et al. 2023. “Molecular Analysis of Broad‐Spectrum Induced Resistance in Rice by the Green Leaf Volatile Z‐ 3 ‐ Hexenyl Acetate.” Journal of Experimental Botany 74: 6804–6819. 37624920 10.1093/jxb/erad338

[pce70279-bib-0010] Dongus, J. A. , and J. E. Parker . 2021. “EDS1 Signalling: At the Nexus of Intracellular and Surface Receptor Immunity.” Current Opinion in Plant Biology 62: 102039. 33930849 10.1016/j.pbi.2021.102039

[pce70279-bib-0011] Du, L. , C. Lu , Z. Wang , L. Zou , Y. Xiong , and Q. Zhang . 2024. “GFAnno: Integrated Method for Plant Flavonoid Biosynthesis Pathway Gene Annotation.” Beverage Plant Research 4: e008. 10.48130/bpr-0023-0041.

[pce70279-bib-0012] Engelberth, J. , H. T. Alborn , E. A. Schmelz , and J. H. Tumlinson . 2004. “Airborne Signals Prime Plants Against Insect Herbivore Attack.” Proceedings of the National Academy of Sciences 101: 1781–1785. 10.1073/pnas.0308037100 PMC341853 14749516

[pce70279-bib-0013] Engelberth, J. , I. Seidl‐Adams , J. C. Schultz , and J. H. Tumlinson . 2007. “Insect Elicitors and Exposure to Green Leafy Volatiles Differentially Upregulate Major Octadecanoids and Transcripts of 12‐Oxo Phytodienoic Acid Reductases in *Zea mays* .” Molecular Plant‐Microbe Interactions 20: 707–716. 17555278 10.1094/MPMI-20-6-0707

[pce70279-bib-0014] Erb, M. 2018. “Volatiles as Inducers and Suppressors of Plant Defense and Immunity—Origins, Specificity, Perception and Signaling.” Current Opinion in Plant Biology 44: 117–121. 29674130 10.1016/j.pbi.2018.03.008

[pce70279-bib-0015] Etterson, J. R. , R. H. Toczydlowski , K. J. Winkler , J. A. Kirschbaum , and T. S. McAulay . 2016. “ *Solidago altissima* Differs With Respect to Ploidy Frequency and Clinal Variation Across the Prairie‐Forest Biome Border in Minnesota.” American Journal of Botany 103, no. 1: 22–32. 26507110 10.3732/ajb.1500146

[pce70279-bib-0016] Feng, K. , X. L. Hou , G. M. Xing , et al. 2020. “Advances in AP2/ERF Super‐Family Transcription Factors in Plant.” Critical Reviews in Biotechnology 40: 750–776. 32522044 10.1080/07388551.2020.1768509

[pce70279-bib-0017] Fitt, G. P. 1989. “The Ecology of Heliothis Species in Relation to Agroecosystems.” Annual Review of Entomology 34: 17–53.

[pce70279-bib-0018] Frost, C. J. , M. C. Mescher , J. E. Carlson , and C. M. De Moraes . 2008. “Plant Defense Priming Against Herbivores: Getting Ready for a Different Battle.” Plant Physiology 146: 818–824. 18316635 10.1104/pp.107.113027 PMC2259053

[pce70279-bib-0019] Frost, C. J. , M. C. Mescher , C. Dervinis , J. M. Davis , J. E. Carlson , and C. M. De Moraes . 2008a. “Priming Defense Genes and Metabolites in Hybrid Poplar by the Green Leaf Volatile Cis‐3‐Hexenyl Acetate.” New Phytologist 180: 722–734. 18721163 10.1111/j.1469-8137.2008.02599.x

[pce70279-bib-0020] Gao, C.‐H. , C. Chen , T. Akyol , et al. 2024. “ggVennDiagram: intuitive Venn Diagram Software Extended.” iMeta 3: 69. 10.1002/imt2.177. PMC10989133 38868514

[pce70279-bib-0021] Garcia, A. , M. E. Santamaria , I. Diaz , and M. Martinez . 2021. “Disentangling Transcriptional Responses in Plant Defense Against Arthropod Herbivores.” Scientific Reports 11: 12996. 34155286 10.1038/s41598-021-92468-6 PMC8217245

[pce70279-bib-0022] Goossens, J. , J. Mertens , and A. Goossens . 2017. “Role and Functioning of bHLH Transcription Factors in Jasmonate Signalling.” Journal of Experimental Botany 68: 1333–1347. 27927998 10.1093/jxb/erw440

[pce70279-bib-0023] Haas, B. J. n.d. TransDecoder. https://github.com/TransDecoder/TransDecoder.

[pce70279-bib-0024] Haas, B. J. , A. Papanicolaou , M. Yassour , et al. 2013. “De Novo Transcript Sequence Reconstruction From RNA‐Seq Using the Trinity Platform for Reference Generation and Analysis.” Nature Protocols 8: 1494–1512. 10.1038/nprot.2013.084. 23845962 PMC3875132

[pce70279-bib-0025] Halverson, K. , S. B. Heard , J. D. Nason , and J. O. Stireman . 2008. “Origins, Distribution, and Local Co‐Occurrence of Polyploid Cytotypes in *Solidago altissima* (Asteraceae).” American Journal of Botany 95: 50–58. 21632314 10.3732/ajb.95.1.50

[pce70279-bib-0026] Han, Z. , and R. Schneiter . 2024. “Dual Functionality of Pathogenesis‐Related Proteins: Defensive Role in Plants Versus Immunosuppressive Role in Pathogens.” Frontiers in Plant Science 15: 1368467. 10.3389/fpls.2024.1368467. 39157512 PMC11327054

[pce70279-bib-0027] Heath, J. J. , A. Kessler , E. Woebbe , D. Cipollini , and J. O. Stireman . 2014. “Exploring Plant Defense Theory in Tall Goldenrod, *Solidago altissima* .” New Phytologist 202: 1357–1370. 24611577 10.1111/nph.12755

[pce70279-bib-0028] Helms, A. M. , C. M. De Moraes , M. C. Mescher , and J. F. Tooker . 2014. “The Volatile Emission of *Eurosta solidaginis* Primes Herbivore‐Induced Volatile Production in *Solidago altissima* and Does Not Directly Deter Insect Feeding.” BMC Plant Biology 14: 173. 24947749 10.1186/1471-2229-14-173 PMC4071026

[pce70279-bib-0029] Helms, A. M. , C. M. De Moraes , J. F. Tooker , and M. C. Mescher . 2013. “Exposure of *Solidago altissima* Plants to Volatile Emissions of an Insect Antagonist (*Eurosta solidaginis*) Deters Subsequent Herbivory.” Proceedings of the National Academy of Sciences 110: 199–204. 10.1073/pnas.1218606110 PMC3538263 23237852

[pce70279-bib-0030] Helms, A. M. , C. M. De Moraes , A. Tröger , et al. 2017. “Identification of an Insect‐Produced Olfactory Cue That Primes Plant Defenses.” Nature Communications 8: 337. 10.1038/s41467-017-00335-8 PMC5569085 28835618

[pce70279-bib-0031] Hilker, M. , and T. Schmülling . 2019. “Stress Priming, Memory, and Signalling in Plants.” Plant, Cell & Environment 42: 753–761. 10.1111/pce.13526 30779228

[pce70279-bib-0032] Hilker, M. , J. Schwachtje , M. Baier , et al. 2016. “Priming and Memory of Stress Responses in Organisms Lacking a Nervous System.” Biological Reviews 91: 1118–1133. 26289992 10.1111/brv.12215

[pce70279-bib-0033] Huerta‐cepas , J. Szklarczyk , D. Heller , et al. 2019. eggNOG 5.0: A Hierarchical, Functionally and Phylogenetically Annotated Orthology Resource Based on 5090 Organisms and 2502 Viruses. 10.1093/nar/gky1085. PMC6324079 30418610

[pce70279-bib-0034] Jin, J. , F. Tian , D. C. Yang , et al. 2017. “PlantTFDB 4.0: Toward a Central Hub for Transcription Factors and Regulatory Interactions in Plants.” Nucleic Acids Research 45: D1040–D1045. 27924042 10.1093/nar/gkw982 PMC5210657

[pce70279-bib-0035] Karban, R. 2011. “The Ecology and Evolution of Induced Resistance Against Herbivores.” Functional Ecology 25: 339–347.

[pce70279-bib-0036] Karssemeijer, P. N. , K. A. de Kreek , R. Gols , et al. 2022. “Specialist Root Herbivore Modulates Plant Transcriptome and Downregulates Defensive Secondary Metabolites in a Brassicaceous Plant.” New Phytologist 235: 2378–2392. 35717563 10.1111/nph.18324 PMC9540780

[pce70279-bib-0037] Kessler, A. , and M. B. Mueller . 2024. “Induced Resistance to Herbivory and the Intelligent Plant.” Plant Signaling & Behavior 19, no. 1: 2345985. 10.1080/15592324.2024.2345985. 38687704 PMC11062368

[pce70279-bib-0038] Kim, J. , and G. W. Felton . 2013. “Priming of Antiherbivore Defensive Responses in Plants.” Insect Science 20: 273–285. 23955880 10.1111/j.1744-7917.2012.01584.x

[pce70279-bib-0039] Kim, J. , J. F. Tooker , D. S. Luthe , C. M. De Moraes , and G. W. Felton . 2012. “Insect Eggs Can Enhance Wound Response in Plants: A Study System of Tomato *solanum lycopersicum* L. and *Helicoverpa zea* Boddie.” PLoS One 7: e37420. 22616005 10.1371/journal.pone.0037420 PMC3352884

[pce70279-bib-0040] Langfelder, P. , and S. Horvath . 2008. “WGCNA: An R Package for Weighted Correlation Network Analysis.” BMC Bioinformatics 9: 559. 19114008 10.1186/1471-2105-9-559 PMC2631488

[pce70279-bib-0041] Lawson, S. K. , L. G. Sharp , C. N. Powers , R. L. McFeeters , P. Satyal , and W. N. Setzer . 2020. “Volatile Compositions and Antifungal Activities of Native American Medicinal Plants: Focus on the Asteraceae.” Plants 9, no. 1: 126. 10.3390/plants9010126. 31963839 PMC7020142

[pce70279-bib-0042] Li, C. , W. Zha , W. Li , J. Wang , and A. You . 2023. “Advances in the Biosynthesis of Terpenoids and Their Ecological Functions in Plant Resistance.” International Journal of Molecular Sciences 24: 11561. 37511319 10.3390/ijms241411561 PMC10380271

[pce70279-bib-0043] Liu, M. , Z. Su , S. Zhang , et al. 2025. “A Secreted Glycerophosphodiester Phosphodiesterase (GDPD) Domain—Containing Protein, FpGDPD, Is Involved in Fusarium Pseudograminearum Virulence.” Mycologia 117: 925–936. 40569204 10.1080/00275514.2025.2516372

[pce70279-bib-0044] Liu, Q. , Q. Fu , Y. Yan , et al. 2024. “Curation, Nomenclature, and Topological Classification of Receptor‐Like Kinases From 528 Plant Species for Novel Domain Discovery and Functional Inference.” Molecular Plant 17: 658–671. 38384130 10.1016/j.molp.2024.02.015

[pce70279-bib-0046] Liu, Y. , A. Beyer , and R. Aebersold . 2016. “On the Dependency of Cellular Protein Levels on mRNA Abundance.” Cell 165: 535–550. 27104977 10.1016/j.cell.2016.03.014

[pce70279-bib-0047] Love, M. I. , W. Huber , and S. Anders . 2014. “Moderated Estimation of Fold Change and Dispersion for RNA‐Seq Data With DESeq. 2.” Genome Biology 15: 550. 10.1186/s13059-014-0550-8. 25516281 PMC4302049

[pce70279-bib-0048] Machado, R. A. R. , C. A. M. Robert , C. C. M. Arce , et al. 2016. “Auxin Is Rapidly Induced by Herbivore Attack and Regulates a Subset of Systemic, Jasmonate‐Dependent Defenses.” Plant Physiology 172: 521–532. 27485882 10.1104/pp.16.00940 PMC5074610

[pce70279-bib-0049] MacIntosh, G. C. 2011. “RNase T2 Family: Enzymatic Properties, Functional Diversity, and Evolution of Ancient Ribonucleases.” In Ribonucleases, edited by A. W. Nicholson , 89–114. Springer.

[pce70279-bib-0050] Magalhães, D. M. , I. T. F. A. Da Silva , M. Borges , R. A. Laumann , and M. C. Blassioli‐moraes . 2019. “Anthonomus Grandis Aggregation Pheromone Induces Cotton Indirect Defence and Attracts the Parasitic Wasp *Bracon vulgaris* .” Journal of Experimental Botany 70, no. 6: 1891–1901. 10.1093/jxb/erz040. 30722044

[pce70279-bib-0052] Manni, M. , M. R. Berkeley , M. Seppey , and E. M. Zdobnov 2021. “BUSCO: Assessing Genomic Data Quality and Beyond.” *Current Protocols* 1: e323. 10.1002/cpz1.323. 34936221

[pce70279-bib-0053] Martinez‐Medina, A. , V. Flors , M. Heil , et al. 2016. “Recognizing Plant Defense Priming.” Trends in Plant Science 21: 818–822. 27507609 10.1016/j.tplants.2016.07.009

[pce70279-bib-0054] Mauch‐Mani, B. , I. Baccelli , E. Luna , and V. Flors . 2017. “Defense Priming: An Adaptive Part of Induced Resistance.” Annual Review of Plant Biology 68: 485–512. 10.1146/annurev-arplant-042916-041132 28226238

[pce70279-bib-0055] Montesinos, Á. , S. Sacristán , P. del Prado‐Polonio , et al. 2024. “Contrasting Plant Transcriptome Responses Between a Pierce‐Sucking and a Chewing Herbivore Go Beyond the Infestation Site.” BMC Plant Biology 24: 120. 38369495 10.1186/s12870-024-04806-1 PMC10875829

[pce70279-bib-0056] Nuruzzaman, M. , A. M. Sharoni , and S. Kikuchi . 2013. “Roles of NAC Transcription Factors in the Regulation of Biotic and Abiotic Stress Responses in Plants.” Frontiers in Microbiology 4: 1–16. 24058359 10.3389/fmicb.2013.00248 PMC3759801

[pce70279-bib-0057] Oliva, M. L. V. , M. C. C. Silva , R. C. Sallai , M. V. Brito , and M. U. Sampaio . 2010. “A Novel Subclassification for Kunitz Proteinase Inhibitors From Leguminous Seeds.” Biochimie 92: 1667–1673. 20363284 10.1016/j.biochi.2010.03.021

[pce70279-bib-0058] Pagès, H. , M. Carlson , S. Falcon , and N. Li 2023. AnnotationDbi: Manipulation of SQLite‐Based Annotations in Bioconductor. 10.18129/B9.bioc.AnnotationDbi.

[pce70279-bib-0059] Pan, L. , R. Huang , Z. Lu , et al. 2024. “Combined Transcriptome and Metabolome Analysis Identifies Triterpenoid‐Induced Defense Responses in *Myzus persicae* Sülzer‐Infested Peach.” Journal of Experimental Botany 75: 6644–6662. 39110720 10.1093/jxb/erae339

[pce70279-bib-0060] Pantano, L. 2017. *DEGreport: Report of DEG Analysis*. R Package Version 1.13.8. https://bioconductor.org/packages/DEGreport.

[pce70279-bib-0061] Petrussa, E. , E. Braidot , M. Zancani , et al. 2013. “Plant Flavonoids — Biosynthesis, Transport and Involvement in Stress Responses.” International Journal of Molecular Sciences 14: 14950–14973. 23867610 10.3390/ijms140714950 PMC3742282

[pce70279-bib-0062] Priya, P. , A. Yadav , J. Chand , and G. Yadav . 2018. “Terzyme: A Tool for Identification and Analysis of the Plant Terpenome.” Plant Methods 14: 4. 29339971 10.1186/s13007-017-0269-0 PMC5761147

[pce70279-bib-0063] R Core Team . 2023. R: A Language and Environment for Statistical Computing. R Foundation for Statistical Computing, Vienna, Austria. https://www.R-project.org/.

[pce70279-bib-0064] Ren, C. , J. Qian , Y. Wang , et al. 2025. “Flavonoid UDP‐Glycosyltransferase in Plants: Functional Identification, Substrate Recognition Mechanism, and Biotechnology Application.” Phytochemistry Reviews 24: 4451–4474. 10.1007/s11101-024-10042-0.

[pce70279-bib-0065] Ruan, J. , Y. Zhou , M. Zhou , et al. 2019. “Jasmonic Acid Signaling Pathway in Plants.” International Journal of Molecular Sciences 20: 2479. 31137463 10.3390/ijms20102479 PMC6566436

[pce70279-bib-1066] Santamaría, M. E. , M. Diaz‐Mendoza , M. Diaz , and I Martinez . 2014. “Plant Protein Peptidase Inhibitors: An Evolutionary Overview Based on Comparative Genomics.” BMC Genomics 15: 812. 25253557 10.1186/1471-2164-15-812 PMC4189545

[pce70279-bib-0066] Schmelz, E. A. , J. Engelberth , J. H. Tumlinson , A. Block , and H. T. Alborn . 2004. “The Use of Vapor Phase Extraction in Metabolic Profiling of Phytohormones and Other Metabolites.” Plant Journal 39: 790–808. 10.1111/j.1365-313X.2004.02168.x 15315639

[pce70279-bib-0067] Schwanhäusser, B. , D. Busse , N. Li , et al. 2011. “Global Quantification of Mammalian Gene Expression Control.” Nature 473: 337–342. 21593866 10.1038/nature10098

[pce70279-bib-0068] Schweizer, F. , N. Bodenhausen , S. Lassueur , F. G. Masclaux , and P. Reymond . 2013. “Differential Contribution of Transcription Factors to *Arabidopsis thaliana* Defense Against *Spodoptera littoralis* .” Frontiers in Plant Science 4: 1–12. 23382734 10.3389/fpls.2013.00013 PMC3563046

[pce70279-bib-0069] Stroud, E. A. , J. Jayaraman , M. D. Templeton , and E. H. A. Rikkerink . 2022. “Comparison of the Pathway Structures Influencing the Temporal Response of Salicylate and Jasmonate Defence Hormones in *Arabidopsis thaliana* .” Frontiers in Plant Science 13: 1–20. 10.3389/fpls.2022.952301 PMC9504473 36160984

[pce70279-bib-0070] Ton, J. , M. D'Alessandro , V. Jourdie , et al. 2007. “Priming by Airborne Signals Boosts Direct and Indirect Resistance in Maize.” Plant Journal 49: 16–26. 10.1111/j.1365-313X.2006.02935.x 17144894

[pce70279-bib-0071] Tzin, V. , Y. Hojo , S. R. Strickler , et al. 2017. “Rapid Defense Responses in Maize Leaves Induced by *Spodoptera exigua* Caterpillar Feeding.” Journal of Experimental Botany 68: 4709–4723. 28981781 10.1093/jxb/erx274 PMC5853842

[pce70279-bib-0072] Uhler, L. D. 1951. Biology and Ecology of the Goldenrod Gall Fly, *Eurosta solidaginis* (Fitch). Cornell University.

[pce70279-bib-0073] Vos, I. A. , A. Verhage , R. C. Schuurink , L. G. Watt , C. M. J. Pieterse , and S. C. M. Van Wees . 2013. “Onset of Herbivore‐Induced Resistance in Systemic Tissue Primed for Jasmonate‐Dependent Defenses Is Activated by Abscisic Acid.” Frontiers in Plant Science 4: 1–10. 24416038 10.3389/fpls.2013.00539 PMC3874679

[pce70279-bib-0074] Wang, X. , C. Tang , L. Deng , et al. 2010. “Characterization of a Pathogenesis‐Related Thaumatin‐Like Protein Gene TaPR5 From Wheat Induced by Stripe Rust Fungus.” Physiologia Plantarum 139: 27–38. 20059734 10.1111/j.1399-3054.2009.01338.x

[pce70279-bib-0076] Xu, S. , E. Hu , Y. Cai , et al. 2024. “Using clusterProfiler to Characterize Multiomics Data.” Nature Protocols 19: 3292–3320. 39019974 10.1038/s41596-024-01020-z

[pce70279-bib-0077] Ye, M. , G. Glauser , Y. Lou , M. Erb , and L. Hu . 2019. “Molecular Dissection of Early Defense Signaling Underlying Volatile‐Mediated Defense Regulation and Herbivore Resistance in Rice.” Plant Cell 31: 687–698. 30760558 10.1105/tpc.18.00569 PMC6482627

[pce70279-bib-0078] Yip, E. C. , M. C. Mescher , C. M. De Moraes , and J. F. Tooker . 2025. “An Insect Pheromone Primes Tolerance of Herbivory in Goldenrod Plants.” Ecology 106: 1–8. 39608409 10.1002/ecy.4486

[pce70279-bib-0079] Yip, E. C. , C. M. De Moraes , M. C. Mescher , and J. F. Tooker . 2017. “The Volatile Emission of a Specialist Herbivore Alters Patterns of Plant Defence, Growth and Flower Production in a Field Population of Goldenrod.” Functional Ecology 31: 1062–1070.

[pce70279-bib-0080] Yip, E. C. , C. M. De Moraes , J. F. Tooker , and M. C. Mescher . 2021. “Sensory Co‐Evolution: The Sex Attractant of a Gall‐Making Fly Primes Plant Defences, but Female Flies Recognize Resulting Changes in Host‐Plant Quality.” Journal of Ecology 109: 99–108.

[pce70279-bib-0081] Zhang, X. , W. Ran , J. Zhang , et al. 2020. “Genome‐Wide Identification of the Tify Gene Family and Their Expression Profiles in Response to Biotic and Abiotic Stresses in Tea Plants (*Camellia sinensis*).” International Journal of Molecular Sciences 21: 8316. 33167605 10.3390/ijms21218316 PMC7664218

