# Supplementary material for: A Volatile Cue From a Specialist Herbivore Primes Gene Expression Against Biotic Stress in Tall Goldenrod (Solidago altissima L.)
Source: Plant Cell Environ. 2025 Nov 30;49(3):1424–38. doi: 10.1111/pce.70279 (PMC12873530; doi:10.1111/pce.70279)
Supplement: Supplementary file 1 — Supplementary Figure S1: Schematic of experimental design. [file PCE-49-1424-s007.pdf]

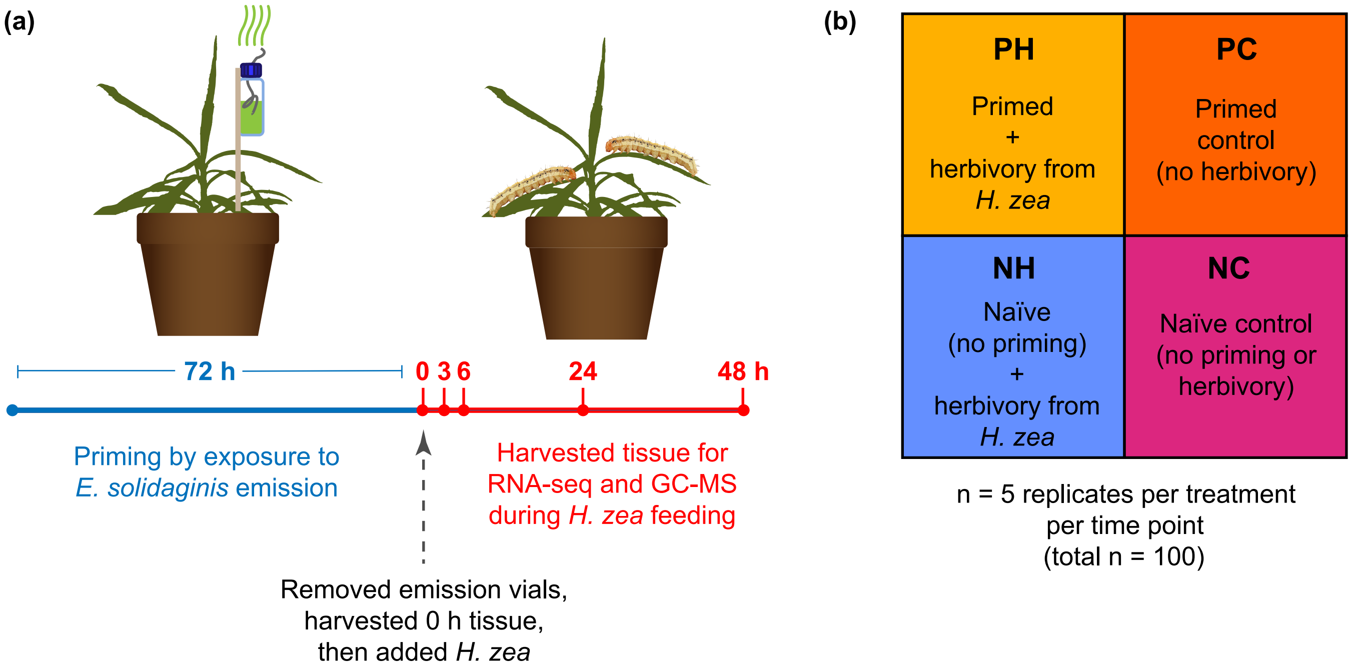
(a)
72 h
0 3 6
24
48 h
Priming by exposure to
E. solidaginis emission
Harvested tissue for
RNA-seq and GC-MS
during *H. zea* feeding
Removed emission vials,
harvested 0 h tissue,
then added *H. zea*
(b)
PH
Primed
+
herbivory from
H. zea
PC
Primed
control
(no herbivory)
NH
Naïve
(no priming)
+
herbivory from
H. zea
NC
Naïve control
(no priming or
herbivory)
n = 5 replicates per treatment
per time point
(total n = 100)
