# Supplementary figures and images for: A Volatile Cue From a Specialist Herbivore Primes Gene Expression Against Biotic Stress in Tall Goldenrod (Solidago altissima L.)
Source: Plant Cell Environ. 2025 Nov 30;49(3):1424–38. doi: 10.1111/pce.70279 (PMC12873530; doi:10.1111/pce.70279)

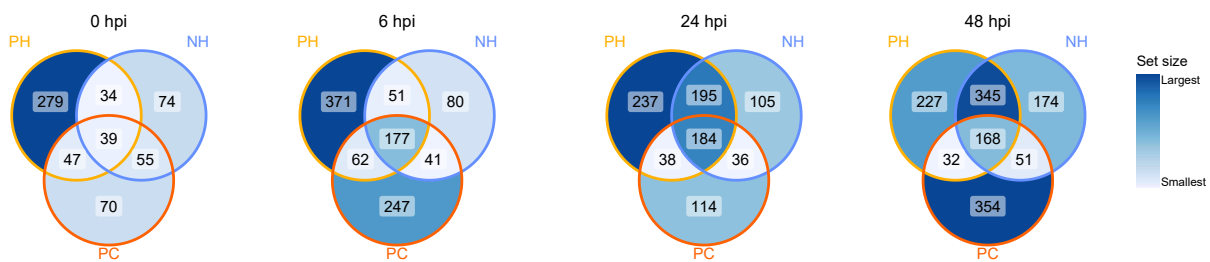

Supplement: Supplementary file 2 — Supplementary Figure S2: Venn diagrams of downregulated DEGs. [file PCE-49-1424-s001.pdf]

All upregulated DEGs (n = 2,650) clustered by time

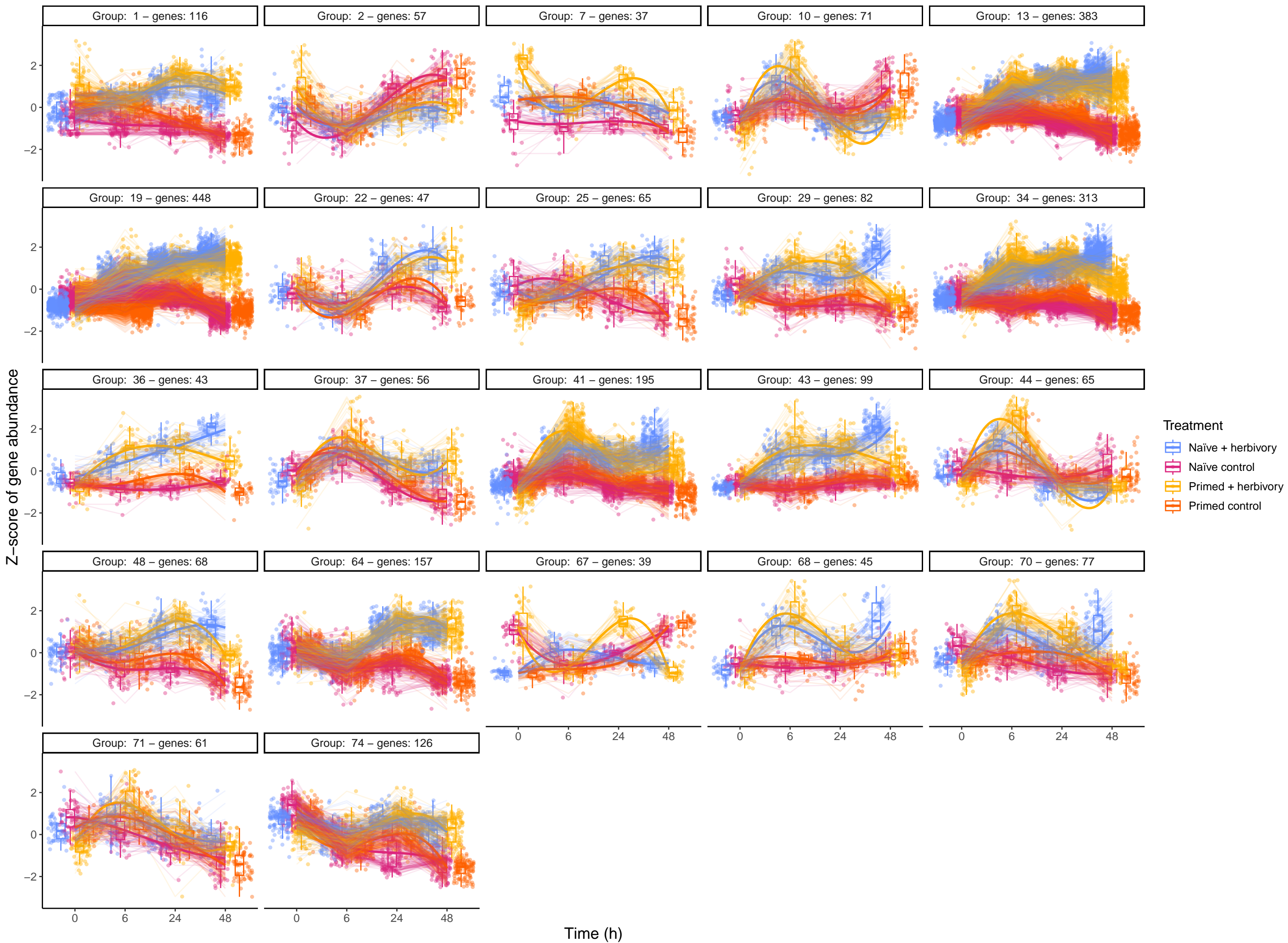

Supplement: Supplementary file 3 — Supplementary Figure S3: All time‐based DEG clusters produced in DEGreport. [file PCE-49-1424-s009.pdf]

**(a)** Defense-related Transcription factors (n = 66)

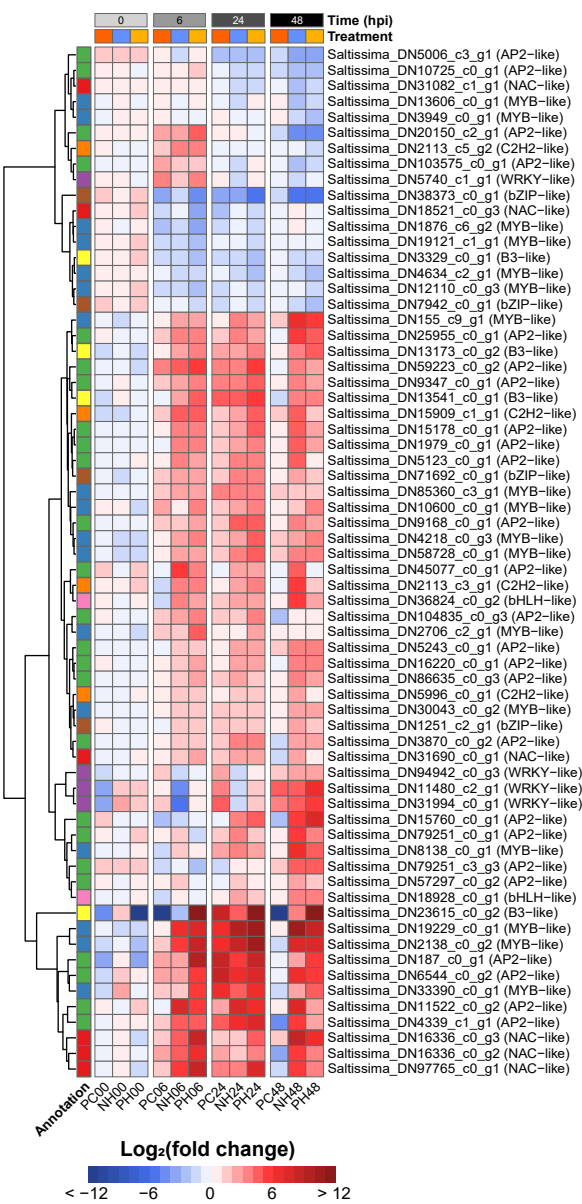

**(b)** Defense-related Receptor-like kinases (n = 60)

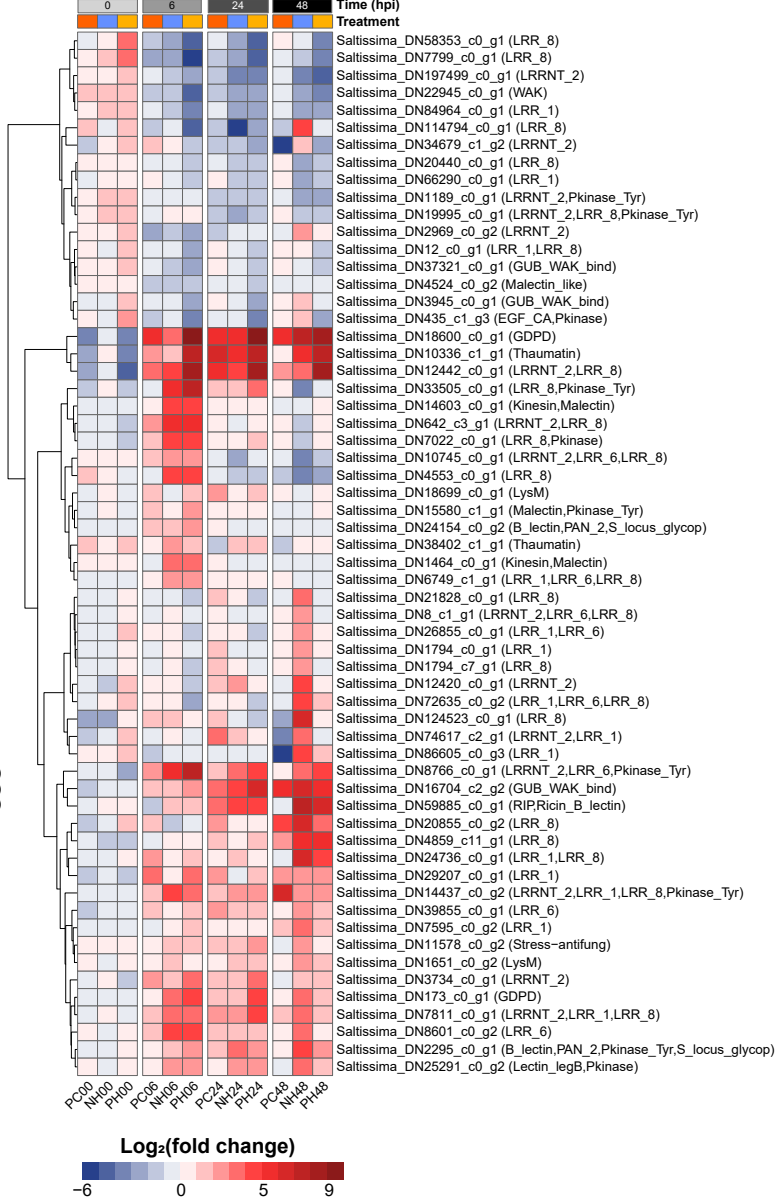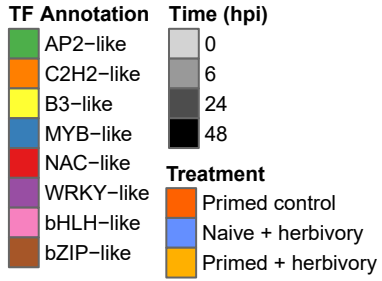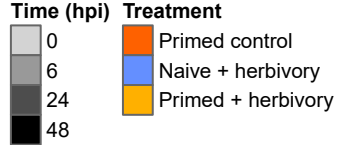

Supplement: Supplementary file 4 — Supplementary Figure S4: Expression of receptor‐like kinase and defense‐related transcription factor genes. [file PCE-49-1424-s004.pdf]

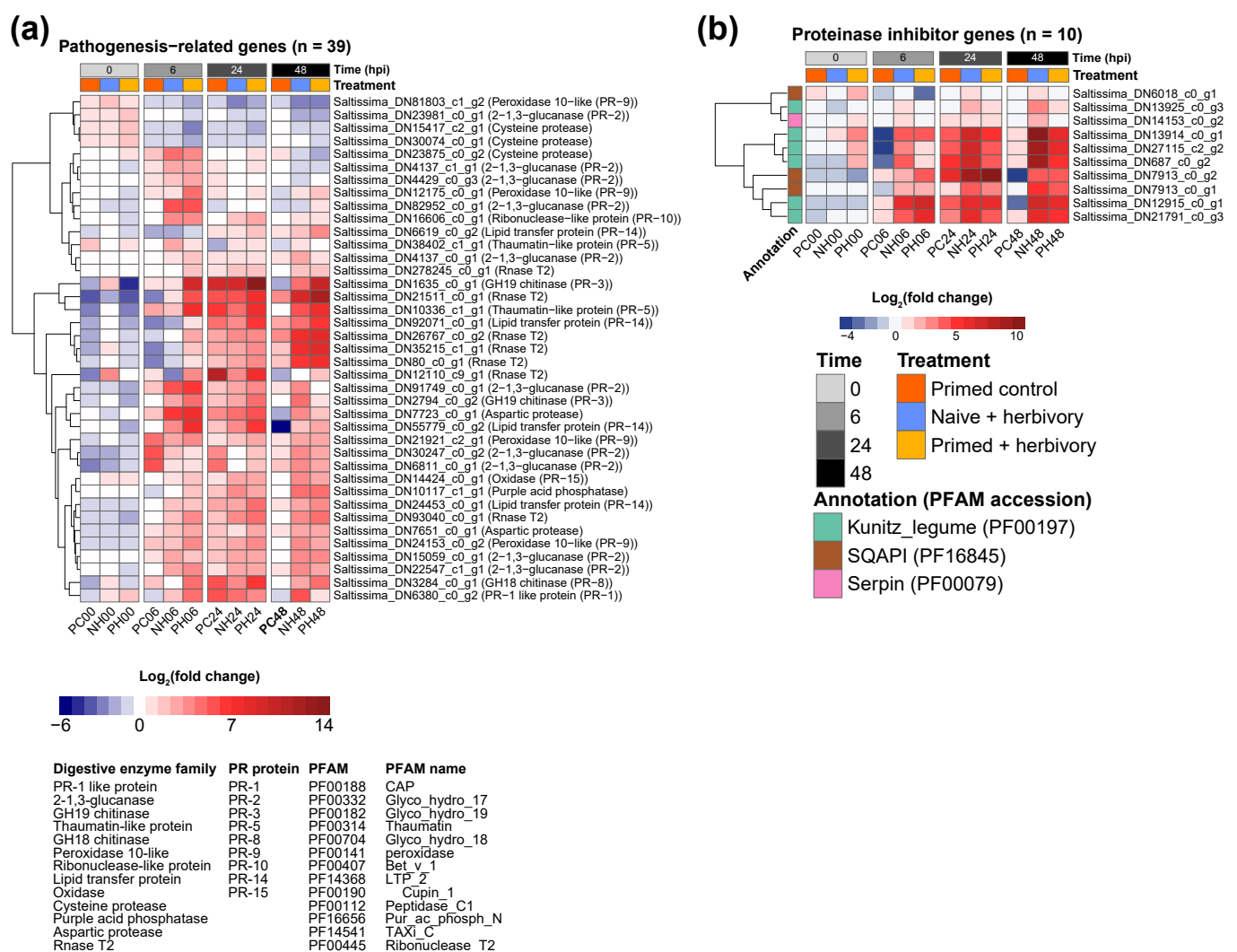

Supplement: Supplementary file 5 — Supplementary Figure S5. Expression of pathogenesis‐related and proteinase inhibitor genes. [file PCE-49-1424-s005.pdf]

(a)

## Terpene biosynthesis genes n = 18

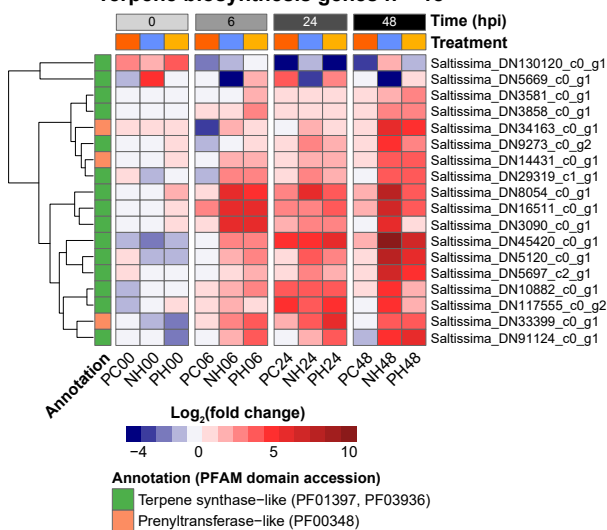

(b)

## Flavonoid biosynthesis genes n=39

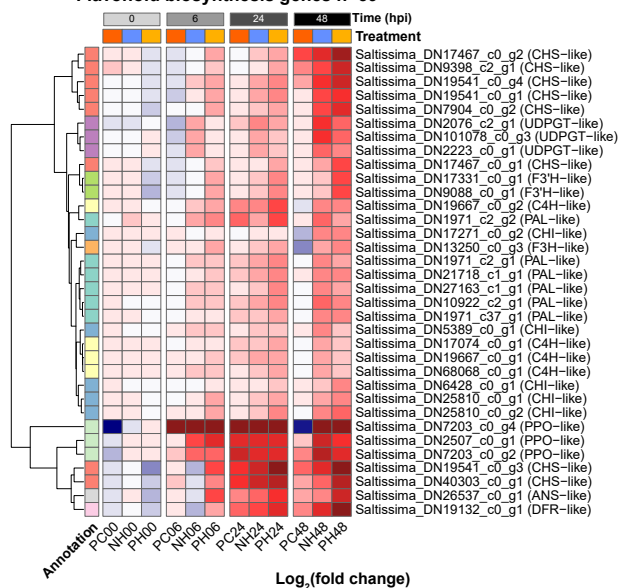

(c)

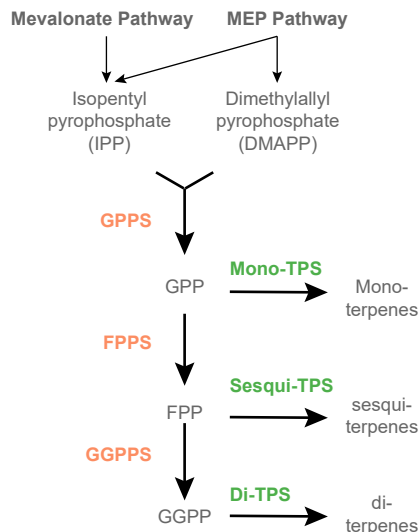

(d)

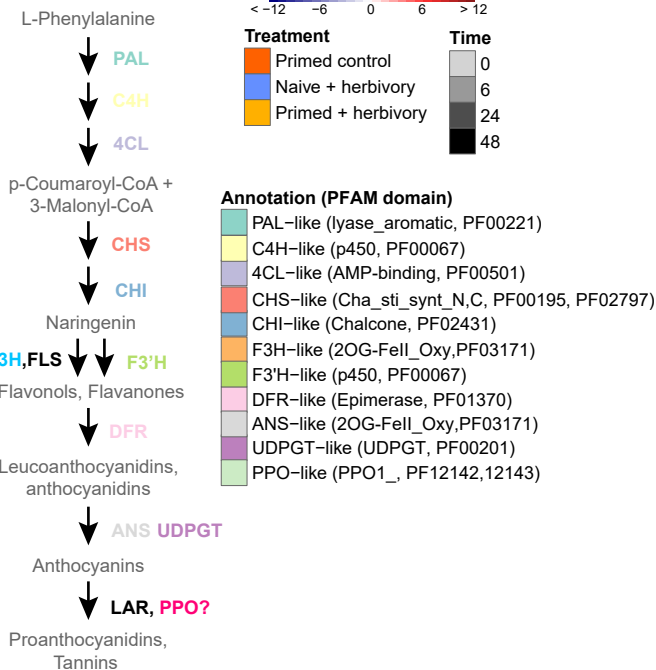

Supplement: Supplementary file 6 — Supplementary Figure S6: Expression of genes in the terpenoid and flavonoid biosynthesis pathways. [file PCE-49-1424-s006.pdf]

(a)

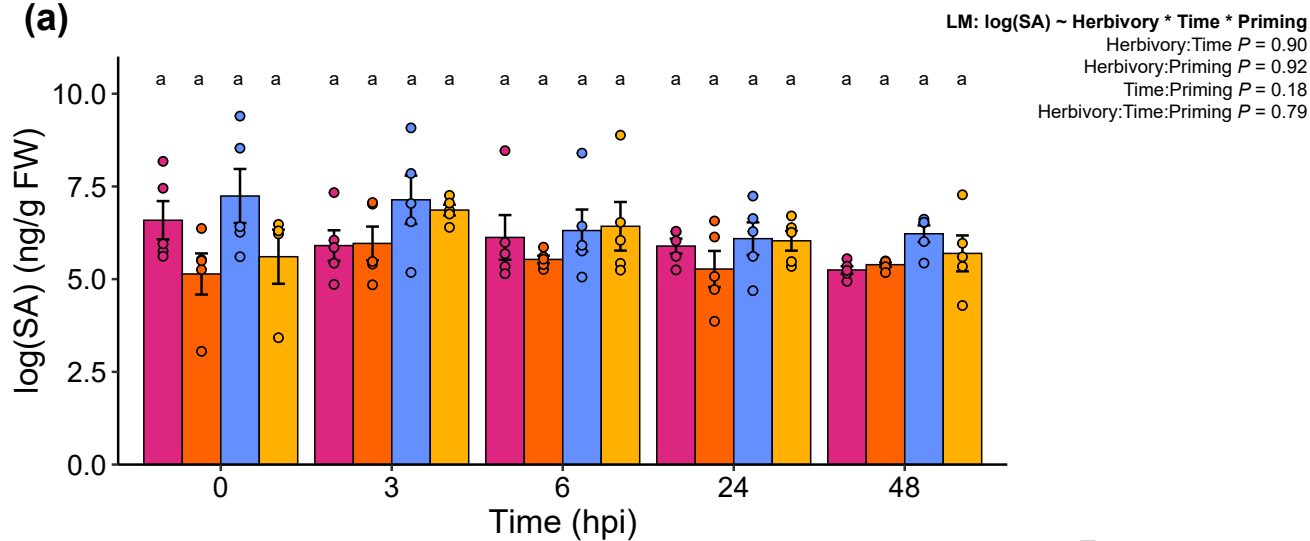

(b)

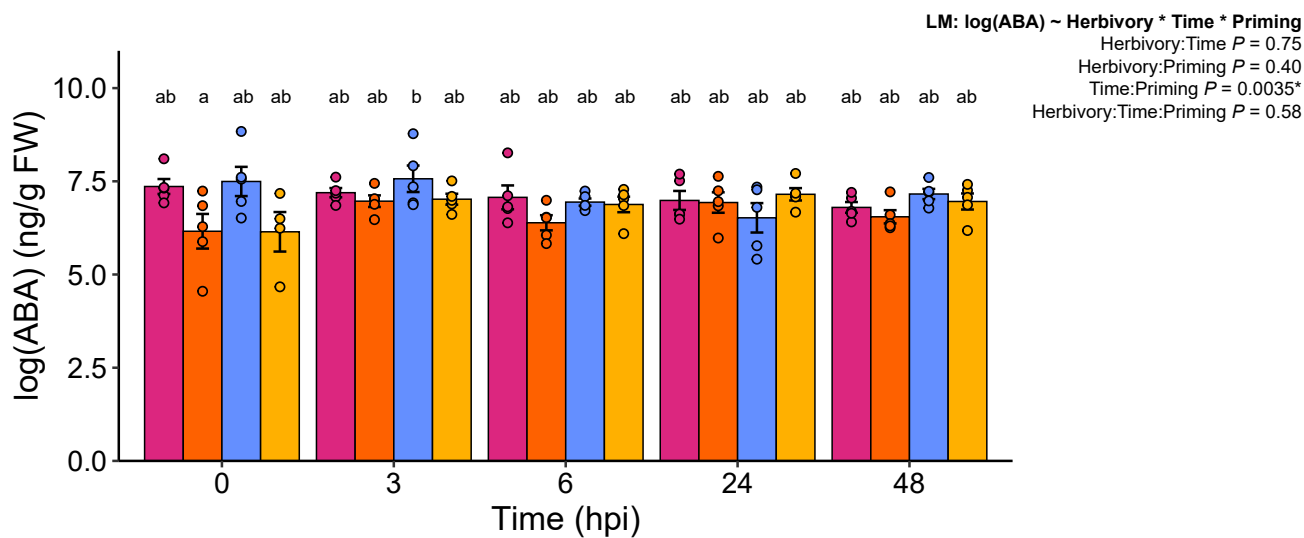

Supplement: Supplementary file 7 — Supplementary Figure S7: Levels of salicylic acid and abscisic acid. [file PCE-49-1424-s002.pdf]
