# Supplementary material for: A Volatile Cue From a Specialist Herbivore Primes Gene Expression Against Biotic Stress in Tall Goldenrod (Solidago altissima L.)
Source: Plant Cell Environ. 2025 Nov 30;49(3):1424–38. doi: 10.1111/pce.70279 (PMC12873530; doi:10.1111/pce.70279)
Supplement: Supplementary file 9 — Supmat. [file PCE-49-1424-s008.docx]

**Supporting Information**

**Supplementary Figure S1.** Schematic of experimental design.

**Supplementary Figure S2.** Venn diagrams of downregulated DEGs.

**Supplementary Figure S3.** All time-based DEG clusters produced in DEGreport.

**Supplementary Figure S4.** Expression of receptor-like kinase and defense-related transcription factor genes.

**Supplementary Figure S5.** Expression of pathogenesis-related and proteinase inhibitor genes.

**Supplementary Figure S6.** Expression of genes in the terpenoid and flavonoid biosynthesis pathways.

**Supplementary Figure S7.** Levels of salicylic acid and abscisic acid.

**Supplementary Table S1.** Transcriptome assembly statistics.

**Supplementary Table S2.** Transcriptome annotation hits generated in eggNOGmapper.

**Supplementary Table S3.** Differentially expressed genes for all 12 pairwise comparisons.

**Supplementary Table S4.** DEGs in all hierarchical clusters

**Supplementary Table S5.** Gene ontology enrichment for all DEG clusters.

**Supplementary Table S6.** Venn diagram sets of shared DEGs.

**Supplementary Table S7.** Gene ontology enrichment for all shared DEG sets.
